# Coulombic-enhanced hetero radical pairing interactions

**DOI:** 10.1038/s41467-018-04335-0

**Published:** 2018-05-17

**Authors:** Xujun Zheng, Yang Zhang, Ning Cao, Xin Li, Shuoqing Zhang, Renfeng Du, Haiying Wang, Zhenni Ye, Yan Wang, Fahe Cao, Haoran Li, Xin Hong, Andrew C.-H. Sue, Chuluo Yang, Wei-Guang Liu, Hao Li

**Affiliations:** 1 Department of Chemistry, Zhejiang University, 310027 Hangzhou, China; 2 Department of Chemistry, Hubei Collaborative Innovation Center for Advanced Organic Chemical Materials, Hubei Key Lab on Organic and Polymeric Optoelectronic Materials, Wuhan University, 430072 Wuhan, China; 3 Institute for Molecular Design and Synthesis, School of Pharmaceutical Science & Technology, Health Science Platform, Tianjin University, 92 Weijin Road, Nankai District, 300072 Tianjin, China; 4 Department of Chemistry, Chemical Theory Center, and Minnesota Supercomputing Institute, University of Minnesota, Minneapolis, MN 55455-0431 USA

## Abstract

Spin–spin interactions between two identical aromatic radicals have been studied extensively and utilized to establish supramolecular recognition. Here we report that spin-pairing interactions could also take place between two different π-electron radicals, namely a bipyridinium radical cation (**BPY**^+•^) and a naphthalene-1,8:4,5-bis(dicarboximide) radical anion (**NDI**^─•^). The occurrence of this type of previously unreported hetero radical-pairing interactions is attributed to enhancement effect of Coulombic attraction between these two radicals bearing opposite charges. The Coulombic-enhanced hetero radical pairing interactions are employed to drive host–guest recognition, as well as the reversible switching of a bistable [2]rotaxane.

## Abstract (web summary)

Homo radical spin-pairing interactions between two identical aromatic radicals are common in supramolecular chemistry, but hetero interactions between two different aromatic radicals are seldom observed. Here, the authors find that hetero radical pairing between a radical cation and a radical anion, together with Coulombic attraction, can drive host-guest recognition, representing a new supramolecular recognition motif.

## Introduction

**Fig. 1:**
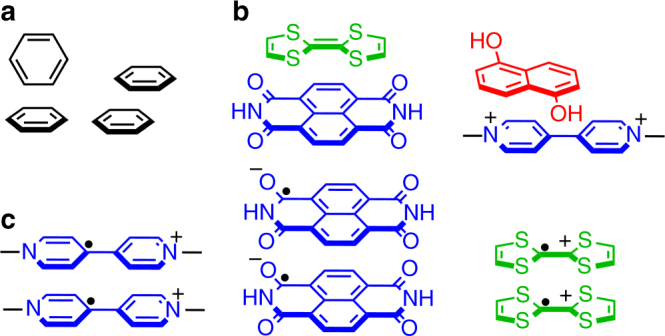
Three types of π-electron interactions. **a** π–π interactions between two benzene rings, in the form of edge-to-face or parallel displaced stacking interactions. **b** Donor–acceptor interactions between **TTF** and **NDI**, as well as **DNP** and **BPY**^2+^. **c** Hom radial paring interactions between two identical radicals, including **BPY**^+•^, **NDI**^─•^, and **TTF**^+•^

It has been known for several decades that aromatic compounds can undergo strong attractive interactions with each other by overlapping their π orbitals. These noncovalent interactions play an important role in the formation of a variety supramolecular architectures in both biological and artificial systems, including stabilizing DNA double helical strands through stacking of base pairs^1^, forming folded tertiary structures of proteins^2^, and host–guest recognition^3,4^, as well as π-electron aggregation for developing organic conductors and semiconductors^5^. In terms of a benzene ring, partial positive and negative charges are distributed within the σ-framework and above the benzene core, respectively. As a consequence, π-electron interactions between two benzene rings often occur in the form of edge-to-face or parallel displaced stacking interactions^6^ (Fig. 1a), in order to maximize π–σ attractions and minimize π–π repulsion. In this case, π-electron interactions are essentially not different from dipole–dipole interactions. When two aromatic moieties contain electron donating and withdrawing functional groups, their π-electron interactions could occur in a manner of face-centered stacking (Fig. 1b), owing to (i) efficient orbital overlap between the highest occupied molecular orbital (HOMO) of the former (the electron-rich one, e.g., tetrathiofuvalene (**TTF**) or dioxynaphthalene (**DNP**)) and lowest unoccupied molecular orbital (LUMO) of the latter (the electron-poor one, e.g., naphthalene-1,8:4,5-bis(dicarboximide) (**NDI**) or 4,4-bipyridinium dication (**BPY**^2+^)), and (ii) the electron-withdrawing group on the acceptor, which polarizes the electron density away and therefore suppresses π-electron repulsion. This type of π-electron interactions are often referred as donor–acceptor interactions^7^. π-electron stacking interactions are also observed between two identical aromatic radical compounds^8^ (Fig. 1c), which are often either cationic or anionic. This homoradical dimerization allows the unpaired radical electrons on the two identical singly occupied molecular orbitals (SOMOs) to undergo spin pairing, forming a diamagnetic radical dimer. This interaction is often extremely enthalpically favorable and can dominate the electrostatic repulsion between the two identical charges. This tendency of homo-dimerization is observed between a variety of aromatic radicals, including **BPY**^+•^ radical cation^9^, **NDI**^─•^ radical anion^10^, **TTF**^+•^ radical cation^11^, and others^12–15^. These homoradical interactions have been harnessed to drive host–guest recognition^16–18^ and create molecular switches and machines^19,20^, as well as develop functional materials^17,21,22^ that have electromagnetic, optical, or magnetic properties.

Even though homoradical spin-pairing interactions between two identical aromatic radicals have been studies extensively, the examples that two different aromatic radicals could undergo hetero radical spin pairing to form supramolecular pimers are relative rare. The latter interactions are observed in a variety of radical-involved reaction intermediates. For example, it was reported that tetracyanoethylene radical anion (**TCNE**^─•^) can interact with a methyl vinyl ether radical cation (**MVE**^+•^), forming a radical pair as the reaction intermediate of a zwitterionic cycloaddition reaction^23^. However, hetero radical-pairing interactions are seldom employed to drive supramolecular recognition, especially in solution state. This might result from the fact that overlapping two different SOMOs is not as thermodynamically favorable as overlapping two identical SOMOs. For example, mixing **TTF** and tetracyanoquinodimethane (**TCNQ**) leads to electron transfer from the former to latter, producing a pair of **TTF**^+•^ radical cation and **TCNQ**^─•^ radical anion. In the context of supramolecular recognition, however, these **TTF**^+•^ radical cations and **TCNQ**^─•^ anions prefer to interact with themselves to form homoradical pairs, namely (**TTF**^+•^)_2_ or (**TCNQ**^─•^)_2_, instead of forming a heterodimer (**TTF**^+•^/**TCNQ**^─•^), as inferred from the single crystallographic analysis^24^. Recently, Stoddart et al.^25^ reported that, with the assistance of mechanically interlocked architectures, **BPY**^+•^ radical cation can undergo radical spin–spin interaction with its more conjugated analog, namely 2,7-diazapyrenium radical cation (**DAP**^+•^). However, we are unaware of any other examples that two constitutionally distinct radicals could undergo radical spin–spin interactions to drive supramolecular recognition. We propose that this unlikely occurring hetero radical interaction might happen, if other supramolecular interactions such as Coulombic attractions are introduced and function cooperatively.

Herein, we demonstrate that a **BPY**^+•^ radical cation and an **NDI**^─•^ radical anion could undergo dimerization, when they are covalently connected. This unreported supramolecular interactions result from the marriage of the hetero radical spin–spin interactions and Coulombic attraction between these two aromatic radicals taking opposite charges. This supramolecular driving force is employed to establish the host–guest recognition between an **NDI**^─•^ guest and a cyclobis(paraquat-*p*-phenylene) (**CBPQT**^2(+•)^) host, whose two **BPY**^+•^ residues are able to undergo hetero radical pairing interactions simultaneously with the anionic guest. This interaction also allows us to develop a molecular switch in the form of a bistable [2]rotaxane, which could be switched either chemically or electrochemically. This [2]rotaxane is composed of a **CBPQT**^4+^ ring component that encircles a dumbbell component containing a **DNP** unit and an **NDI** unit. In oxidative conditions, the ring resides on **DNP** station, thanks to π-electron donor–acceptor interactions. Upon reduction of the **CBPQT**^4+^ ring and **NDI** unit to their dicationic diradical and anionic radical form, namely **CBPQT**^2(+•)^ and **NDI**^─•^, respectively, the ring moves to encircle the latter binding station, on account of hetero radical-pairing interactions.

## Results

### Hetero radical pairing in the presence of covalent bonds

**Fig. 2:**
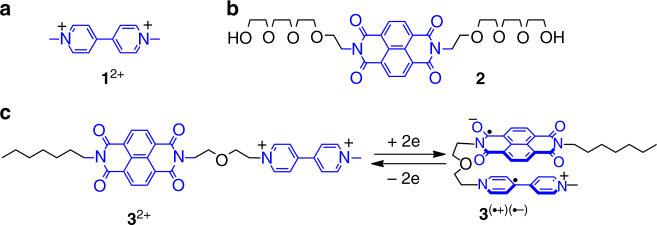
Structural formulas of three model compounds. Structural formulas of (**a**) **1**^2+^, (**b**) **2**, and (**c**) **3**^2+^. Upon reduction of **3**^2+^ to **3**^(+•)(─•)^, the **BPY**^+•^ and **NDI**^─•^ moieties undergo hetero radical-pairing interactions

**Fig. 3:**
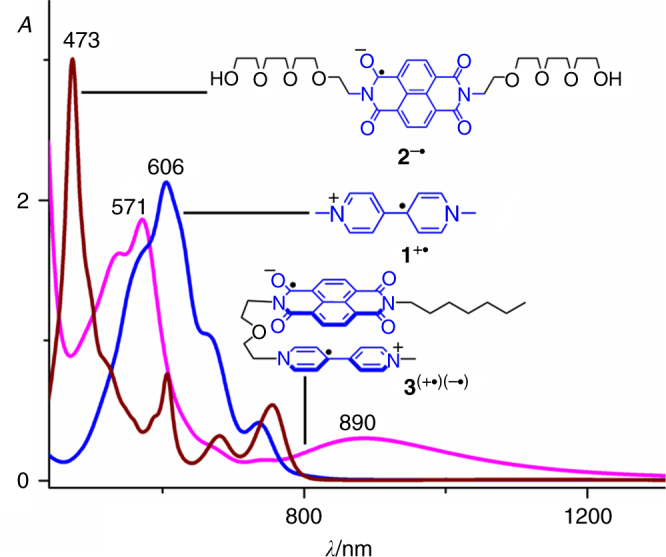
The UV/Vis/NIR absorption studies. The UV/Vis/NIR absorption spectra of **1**^+•^ (blue trace), **2**^─•^ (brown trace), and **3**^(+•)(─•)^ (pink trace) by using cobaltocene as the reducing agent. All UV/Vis/NIR spectra were recorded in a quartz cell with a 10 mm cell path length under the same conditions of temperature (25 °C), solvent (nitrogen-purged MeCN), and concentration (0.1 mM)

We first investigated the possibility of **BPY**^+•^ and **NDI**^─•^ to undergo hetero-dimerization in the absence and presence of covalent connection. Compound **1**^2+^•2PF_6_^─^ and **2** (Fig. 2), which contain either a dicationic **BPY**^2+^ or an **NDI** functional group, respectively, were obtained. Cobaltocene was employed to reduce these two compounds to their radical forms in MeCN, namely **1**^+•^ and **2**^─•^ (PF_6_^─^ anion and cobaltocelium act as the counterions, respectively). The absorption spectra of **1**^+•^ and **2**^─•^ in MeCN were recorded (Fig. 3) by using ultraviolet/visible/near-infrared (UV/Vis/NIR) spectroscopy. As expected, **1**^+•^ and **2**^─•^ have characteristic absorption bands of **BPY**^+•^ and **NDI**^─•^, respectively. The absorption spectrum of the 1:1 mixture of **1**^+•^ and **2**^─•^ is essentially the superimposition of their two individual spectra, indicating that **1**^+•^ and **2**^─•^ does not undergo significant supramolecular interactions (at least not at a concentration of 0.1 mM in MeCN). We then synthesized a compound **3**^2+^•2PF_6_^─^ (Fig. 2) bearing both a **BPY**^2+^ and an **NDI** functional group, which are covalently connected by a glycol chain. Upon reduction, the absorption spectrum of **3**^(+•)(─•)^ was recorded. To our delight, a broadband centered on 890 nm was observed (Fig. 3, pink trace) in the NIR region in the absorption spectrum, accompanied by a blueshifted characteristic absorption band centered on 571 nm, compared to the band centered on 606 nm in the case of **1**^+•^. This result indicates that in the presence of covalent bond that helps to rule out entropy loss, **BPY**^+•^ and **NDI**^─•^ are able to undergo interactions.

**Fig. 4:**
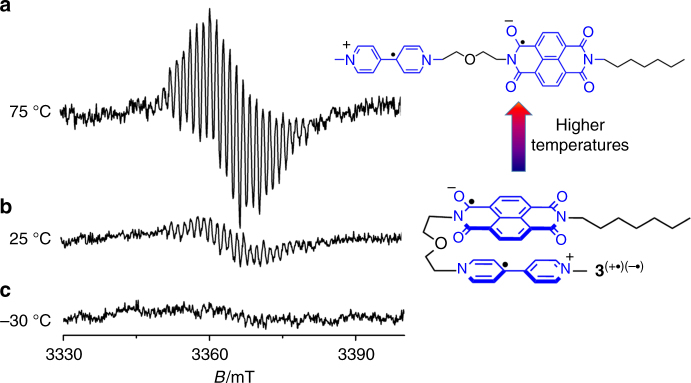
Variable-temperature EPR studies. EPR spectra of **3**^(+•)(─•)^ recorded at (**a**) 75 °C, (**b**) 25 °C, and (**c**) ─30 °C. The concentration of **3**^(+•)(─•)^ is 1 mM. The solvent is nitrogen-purged MeCN. Zink dust was used as the reducing agent, to reduce **3**^2+^ into its radical state, namely **3**^(+•)(─•)^

The electron paramagnetic resonance (EPR) spectrum of **3**^(+•)(─•)^ was recorded (Fig. 4) to justify whether radical spin–spin pairing occurred. It was observed (Fig. 4b) that the EPR signal of **3**^(+•)(─•)^ was significantly weaker, compared to those of **1**^+•^ and **2**^─•^ with the same concentrations in their individual spectra (Supplementary Fig. 14a, b). This observation indicates that within **3**^(+•)(─•)^, the radical electron in **BPY**^+•^ and **NDI**^─•^ underwent spin paring and became diamagnetic to some extent. Addition of **1**^2+^•2PF_6_^─^ into the solution of **3**^(+•)(─•)^ produced (Supplementary Fig. 14c) a characteristic EPR spectrum of **1**^+•^ with high intensity. This observation results from electron transfer form **3**^(+•)(─•)^ to **1**^2+^•2PF_6_^─^, producing **3**^(+•)^ or **3**^2+^ and **1**^+•^, respectively, because **NDI**^─•^ is generally more reductive than **BPY**^+•^. We also performed EPR analysis of **3**^(+•)(─•)^ at variable temperatures ranging from ─30 °C to 75 °C in MeCN. At a higher temperature (e.g., 75 °C), the radical signal underwent a remarkable increase (Fig. 4a). This observation is not surprising because radical pairing between **BPY**^+•^ and **NDI**^─•^ in **3**^(+•)(─•)^ occurred at the expense of entropy loss. As a consequence, at a higher temperature, the **BPY**^+•^/**NDI**^─•^ radical pair undergoes dissociation, increasing its EPR signal. At a lower temperature (─30 °C), when the entropic effect is suppressed, almost no EPR signal is detected (Fig. 4c).

### Hetero radical pairing in host–guest recognition

**CBPQT** ring is a macrocylic host bearing two **BPY** units, which are separated by an approximate 6.8 Å distance on account of the two rigid phenylene bridges^26^. In its fully oxidized state, namely **CBPQT**^4+^, the electron-deficient cavity of the tetracationic host is capable of accommodating a variety of electron-rich guests, such as **TTF** and **DNP**, driven by π-electron donor–acceptor interactions. When the host is reduced to its dicationic diradical state, namely **CBPQT**^2(+•)^, this host was reported^17^ to recognize guests containing BPY^+•^ moieties, owing to homoradical-pairing interactions between the three **BPY**^+•^ radicals in both the host and the guest. The distance (i.e., 6.8 Å) between two **BPY** units in **CBPQT** is twice of efficient π–π distance. This is of importance because host–guest recognition could occur in a manner that the guest is able to undergo π–π interactions simultaneously with each of the two **BPY** units in the host. Encouraged by the result that **BPY**^+•^ and **NDI**^─•^ undergo spin paring when they are covalently connected, we then investigated whether this hetero radical pairing interaction could drive the formation of a supramolecular host–guest complex, namely **NDI**^─•^⊂**CBPQT**^2(+•)^.

**Fig. 5:**
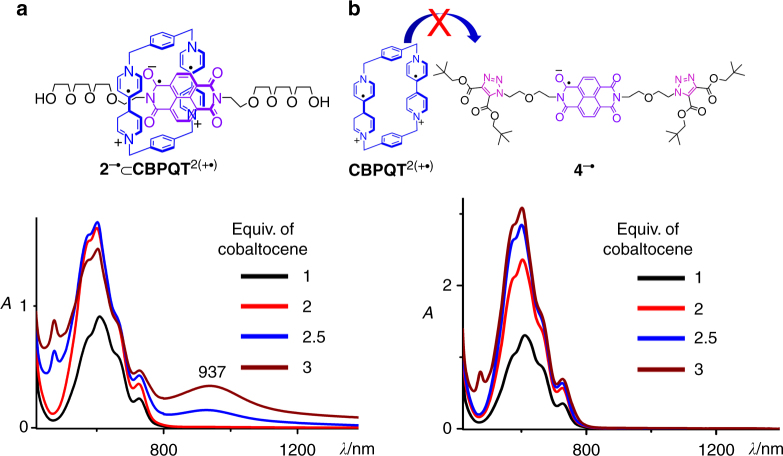
UV/Vis/NIR evidence for host–guest recognition. **a** The UV/Vis/NIR absorption spectra of the 1:1 mixture of **2** (0.1 mM) and **CBPQT**^4+^•4PF_6_^─^ (0.1 mM), after 1 (black trace), 2 (red trace), 2.5 (blue trace), and 3 (brown trace) equivalent cobaltocene was added into the solution. **b** The UV/Vis/NIR absorption spectra of the 1:1 mixture of **4** (0.1 mM) and **CBPQT**^4+^•4PF_6_^─^ (0.1 mM), after 1 (black trace), 2 (red trace), 2.5 (blue trace), and 3 (brown trace) equivalent cobaltocene was added into the solution. All UV/Vis/NIR spectra were recorded in a quartz cell with a 10 mm cell path length under the same conditions of temperature (25 °C), solvent (nitrogen-purged MeCN)

We mixed the **NDI** derirative **2** and **CBPQT**^4+^•4PF_6_^─^ in MeCN in a 1:1 ratio and recorded (Fig. 5a) the absorption spectrum by using UV/Vis spectroscopy. No absorption band was observed in the visible light region, indicating that there are little or no binding affinity between **2** and **CBPQT**^4+^•4PF_6_^─^ in their fully oxidized states, given that both of these two compounds are electron deficient. Cobaltocene was added into the solution to reduce both the host and guest. Addition of no more than two equiv of cobaltocene resulted in the appearance of an absorption band (Fig. 5a) centered at 606 nm, which is the characteristic absorption band of **BPY**^+•^ or the **CBPQT**^2(+•)^ ring, indicating the reduction of the host. Upon addition of more cobaltocene, a broad absorption band centered on 937 nm started to appear (Fig. 5a), whose absorption coefficient reached its maximum after three equiv of cobaltocene was added. This absorption band in the NIR region results from the occurrence of hetero radical pairing interactions between the **NDI**^─•^ in **2**^─•^ and one of the **BPY**^+•^ radicals in the **CBPQT**^2(+•)^ ring, by forming a pseudorotaxane **2**^─•^⊂**CBPQT**^2(+•)^. A dumbbell-shaped compound, namely **4**, containing an **NDI** unit in the middle and two bulky stoppers at each end, was synthesized as a control compound. These two bulky groups are sufficiently large to prevent threading of the ring onto the dumbbell. After adding three equiv of cobaltocene into the 1:1 solution of **4** and **CBPQT**^4+^•4PF_6_^─^ in MeCN, no absorption band in NIR region was observed (Fig. 5b). This result acts as a control experiment, confirming that the binding event between **CBPQT**^2(+•)^ and **2**^─•^ occurs in a manner that the ring threads onto the guest. The so-called alongside interactions between one **BPY**^+•^ unit in **CBPQT**^2(+•)^ ring and **2**^─•^ are not responsible for the occurrence of NIR absorption band, or at least not at this experimental concentration (i.e., 0.1 mM in MeCN). The association constant (*K*_a_) of the 1:1 complex **2**^─•^⊂**CBPQT**^2(+•)^ was measured to be approximate 1.2 ± 0.6 × 10^5^ M^─1^ in MeCN at 298 K, by tracking the absorbance at 937 nm in the UV/Vis/NIR absorption spectra of **CBPQT**^2(+•)^ upon addition of different amount of **2**^─•^ (see Supplementary Fig. 20). A free energy of binding Δ*G* therefore is calculated to be approximate −7 kcal mol^−1^, by using the equation Δ*G* = ─*RT* ln *K*_a_. The formation of **2**^─•^⊂**CBPQT**^2(+•)^ complex was convinced by using EPR spectroscopy (see Supplementary Figs. 15, 16). We also recorded (see Supplementary Fig. 21) the UV/Vis/NIR absorption spectra of the 1:1 complex **2**^─•^⊂**CBPQT**^2(+•)^ at variable temperatures between 283 and 303 K, by which Δ*H* and Δ*S* were determined to be ─7.6 and ─8 cal mol^−1^ K^−1^, respectively.

It is difficult to experimentally quantify the contribution of either Coulombic attraction or hetero radical spin–spin interactions in driving the folding of **3**^(+•)(─•)^ or the formation **2**^─•^⊂**CBPQT**^2(+•)^. This is because it is difficult to obtain a control system that can rule out one of these two supramolecular interactions. We observed that, upon addition of tetrabutylammonium hexafluorophosphate (**TBA**^+^•PF_6_^─^, 50 mM) into the solutions of **2**^─•^⊂**CBPQT**^2(+•)^ in MeCN, the broad absorption band centered on 937 nm disappeared (see Supplementary Fig. 18). This observation indicates that addition of **TBA**^+^•PF_6_^─^ results in dissociation of the **NDI**^─•^⊂**CBPQT**^2(+•)^ complex. This phenomenon could be explained by the fact that Coulombic attraction between **2**^─•^ and **CBPQT**^2(+•)^ was disrupted by **TBA**^+^•PF_6_^─^, which acts as a competitive ionic reagent to interact with the **NDI**^─•^ anions or **CBPQT**^2(+•)^ cations. We thus reasonably hypothesize that Coulombic attraction might play a more important role in their complexation between **2**^─•^ and **CBPQT**^2(+•)^ than radical spin-spin interactions.

Density functional theory (DFT) calculation was performed, in order to better understand the nature of the binding between the two oppositely charged radicals. Theoretical calculation indicates that, center-aligned **BPY**^+•^/**NDI**^─•^ radical pair represents the most thermodynamically optimized state, which has a binding energy (Δ*H*) of ─15.8 kcal mol^−1^, while the energy gap between the triplet and the singlet state of the **BPY**^+•^/**NDI**^─•^ radical dimer is remarkably small, that is, 0.01 kcal mol^─1^ (see Supplementary Fig. 26). The implication is that when the stacking interactions between **BPY**^+•^ and **NDI**^─•^ occur in a center-aligned manner, the major driving force comes from Coulombic attractions. Radical spin–spin interactions do not occur in this case, because the two SOMOs of **BPY**^+•^ and **NDI**^─•^ radicals have mismatched symmetries (see Supplementary Fig. 28a, b). However, when **BPY**^+•^ and **NDI**^─•^ orientate in a parallel displaced manner, the triplet–singlet energy gap becomes larger (Supplementary Fig. 26), implying the occurrence of radical spin–spin interactions. It seems that the center-aligned stacking is more favored than the parallel displaced stacking in terms of binding energy (Δ*H*), because the former orientation maximizes the Coulombic attractions. However, the truth is that, in solution, Coulombic attractions between **BPY**^+•^ and **NDI**^─•^ are often suppressed by solvation or competitive Coulombic interactions from counter-ions, making the parallel displaced stacking more favored. In addition, in the case of **3**^(+•)(─•)^ when a glycol linker is present, the perfect center-aligned orientation of **BPY**^+•^ and **NDI**^─•^ might not be obtainable, also allowing the occurrence of radical spin–spin interactions in the parallel displaced manner.

**Fig. 6:**
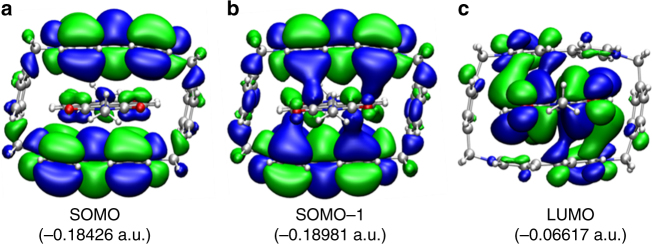
Molecular orbital calculations. Structures (side-view) for **a** singly occupied (SOMO), **b** singly occupied-1 (SOMO-1), and **c** lowest unoccupied (LUMO) molecular orbitals for the inclusion complex **NDI**^─•^⊂**CBPQT**^2(+•)^ complex determined from theoretical calculations. The energy of these three molecular orbital, namely SOMO, SOMO-1, and LUMO, are −0.18426, −0.18981, and −0.06617 a.u., respectively

We also performed DFT calculation to provide (see Fig. 6 and Supplementary Fig. 29) a quantum mechanical description of the **NDI**^─•^⊂**CBPQT**^2(+•)^ complex. The **NDI**^─•^ is partially encapsulated in the cavity of **CBPQT**^2(+•)^, which reflects the fact that fully encapsulating the guest within the host cavity might result in large steric hindrance between the naphthalene protons in the **NDI**^─•^ guest and the phenylene moieties in the **CBPQT**^2(+•)^ host. This partial encapsulation results in a parallel displaced stacking between the **NDI**^─•^ unit in the guest and each of the two **BPY**^+•^ units in the macrocycle, which allows hetero radical spin–spin interactions to occur. The distance between **NDI**^─•^ and each of the two **BPY**^+•^ units of **CBPQT**^2(+•)^ is around 3.3 Å, allowing efficient π–π interactions. By the examination of the frontier molecular orbitals of the radical complex, it has been confirmed that radical spin–spin interactions occurred between the corresponding SOMOs (Fig. 6) of the **NDI**^─•^ and the two **BPY**^+•^ units in the **CBPQT**^2(+•)^ ring, that is, both the SOMO-1 and LUMO orbitals of the complex include significant molecular orbital overlap between **NDI**^─•^ and **CBPQT**^2(+•)^. Natural population analysis^27^ of the **NDI**^─•^⊂**CBPQT**^2(+•)^ complex reveals that the charge on the **NDI**^─•^ and **CBPQT**^2(+•)^ fragments are ─0.96 and +1.96, respectively, indicating occurrence of charge transfer from **NDI**^─•^ to **CBPQT**^2(+•)^.

### Hetero radical pairing in a bistable [2]rotaxane

**Fig. 7:**
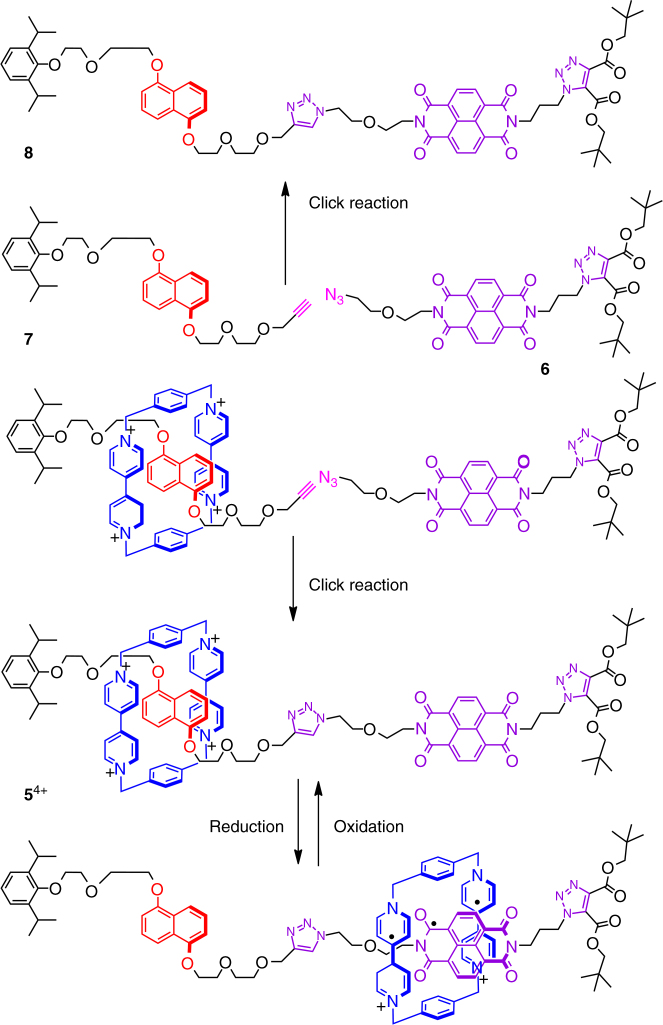
The synthesis and switching of [2]rotaxane 5^4+^. The templated-directed synthesis of the [2]rotaxane **5**^4+^ and its corresponding dumbbell **8**, by means of click reactions. **5**^4+^ could be switched into its reduced state, namely **5**^2(+•)(─•)^, whose diradical dicationic ring component **CBPQT**^2(+•)^ encircles the radical anionic **NDI**^─•^ unit

A bistable [2]rotaxane **5**^4+^•4PF_6_^─^ was designed and synthesized (see Fig. 7 and the details in SI). **5**^4+^•4PF_6_^─^ contains a **CBPQT**^4+^ ring, which encircles a dumbbell bearing a π-electron-rich **DNP** recognition site and a π-electron-deficient **NDI** unit. An **NDI** derivative **6** bearing an azide functional group and a **DNP** derivative **7** containing an alkyne functional group were obtained in relatively high yields in six and four steps, respectively. The bistable [2]rotaxane **5**^4+^•4PF_6_^─^ and its dumbbell counterpart **8** were obtained in 62% and 90% yields, respectively, by means of copper(I)-catalyzed azide–alkyne cycloadditions^28,29^ between **6** and **7** in Me_2_CO in the presence or absence of **CBPQT**^4+^•4PF_6_^─^. The structure of [2]rotaxane **5**^4+^•4PF_6_^─^ was fully characterized by nuclear magnetic resonance (NMR) spectroscopy (see Supplementary Figs. 23, 24) and mass spectrometry (see Supplementary Fig. 25). Both 1D and 2D ^1^H NMR spectra of **5**^4+^•4PF_6_^─^ in CD_3_CN revealed that, in the fully oxidized state, the **CBPQT**^4+^ ring encircles the **DNP** unit in the [2]rotaxane.

**Fig. 8:**
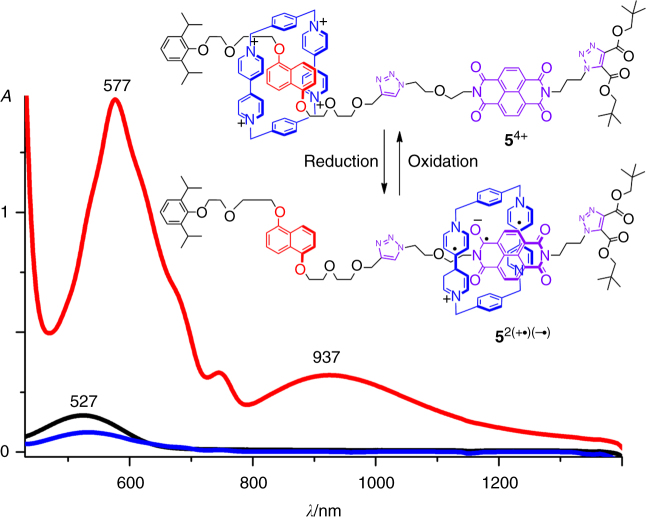
UV/Vis/NIR studies of **5**^4+^. The UV/Vis/NIR absorption spectra of the [2]rotaxane **5**^4+^•4PF_6_^─^ before (black trace) and after (red trace) zinc dust was added into the solution. After exposing the solution to air, the absorption spectra (blue trace) was also recorded. All UV/Vis/NIR spectra were recorded in a quartz cell with a 10 mM cell path length under the same conditions of tempature (25 °C), solvent (nitrogen-purged MeCN), and concentration (0.1 mM)

The switching behavior of **5**^4+^•4PF_6_^─^ by using redox stimuli was monitored (Fig. 8) by using UV/Vis spectroscopy. The absorption 527 nm with a relatively low coefficient was observed (Fig. 8, black trace) before addition of reductant. This characteristic absorption band is on account of the donor–acceptor interactions between the **DNP** unit in the dumbbell and one of the two **BPY**^2+^ unit in the ring, indicating that the ring encircles the **DNP** station. Upon addition of zinc dust into the solution, another characteristic absorption band centered on 577 nm, accompanied by the appearance of a broadband centered on 937 nm, was observed. This observation is consistent with the appearance of the NIR absorption band in the **2**^─•^⊂**CBPQT**^2(+•)^ complex, indicating that after reduction, the **CBPQT**^2(+•)^ ring shuttled and encircled the **NDI**^─•^ station in **5**^2(+•)(─•)^. After exposing the solution to air, the absorption bands centered at 571 and 937 nm were no longer observed, indicating that after oxidation of the ring and the **NDI**^─•^ unit in the dumbbell, the **CBPQT**^4+^ ring moved back and encircling the **DNP** unit.

**Fig. 9:**
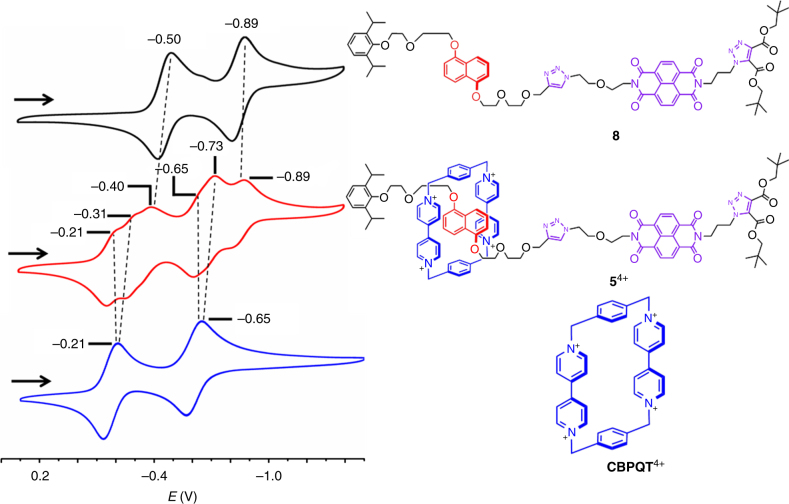
Cyclic voltammetry (CV) studies of **5**^4+^. Second scans of the CVs for the dumbbell **8** (black trace), the [2]rotaxane **5**^4+^•4PF_6_^─^ (red trace), and **CBPQT**^4+^•4PF_6_^─^ (blue trace). All the CVs were recorded under the same conditions of temperature (25 °C), solvent (argon-purged MeCN), concentrations (1 mM), and tetrabutylammonium hexafluorophosphate electrolyte (0.1 M **TBA**^+^•4PF_6_^─^). The scan rate was set at 200 mV s^─1^

The redox-stimulated switching behavior of the [2]rotaxane **5**^4+^•4PF_6_^─^ in MeCN can also be justified by comparing the cyclic voltammetry (CV) data of **5**^4+^•4PF_6_^─^ (Fig. 9, red trace), with those of the **CBPQT**^4+^•4PF_6_^─^ (Fig. 9, blue trace) and the dumbbell **8** (Fig. 9, black trace). The CV of **CBPQT**^4+^•4PF_6_^─^ reveals two consecutive reversible redox processes. The reduction peaks observed at ─0.21 and ─0.65 V can be assigned to two two-electron reductions, namely **CBPQT**^4+^/**CBPQT**^2(+•)^ and **CBPQT**^2(+•)^/**CBPQT**^0^. The CV of the dumbbell **8** also reveals two consecutive reversible redox processes. The reduction peaks observed at ─0.50 and ─0.89 V can be assigned to two one-electron reductions of the **NDI** functional group, namely **NDI**/**NDI**^─•^ and **NDI**^─•^/**NDI**^2─^, respectively. The CV of the [2]rotaxane **5**^4+^•4PF_6_^─^ reveals six reversible redox processes. The first two reduction peaks observed at ─0.21 and ─0.31 V can be assigned to the first two one-electron reduction of the ring, namely **CBPQT**^4+^/**CBPQT**^(+•)(2+)^ and **CBPQT**^(+•)(2+)^/**CBPQT**^2(+•)^. The nonequivalence of the first two reduction potentials of the ring in the [2]rotaxane results from the fact that, in the fully oxidized state of **5**^4+^•4PF_6_^─^, one of the two **BPY**^2+^ units in the ring is more strongly engaged in the π-electron donor–acceptor interactions than the other. The former unit is therefore more difficult to reduce. The third reduction peak of **5**^4+^•4PF_6_^─^ observed at ─0.40 V can be assigned to the one-electron reduction of the **NDI** unit, namely **NDI**/**NDI**^─•^. Compared to the first reduction potential (─0.50 V) of the dumbbell **8**, this reduction peak is shifted to a substantially more positive value by 0.10 V, indicating that the hetero radical-pairing interactions act as the driving force, which makes **NDI** easier to be reduced. This observation supports our assumption that, upon reduction of the [2]rotaxane **5**^4+^•4PF_6_^─^, the **CBPQT**^2(+•)^ ring shuttled and encircled the **NDI**^─•^ unit as the secondary binding station. The fourth reduction peak (─0.65 V) observed for the rotaxane can be assigned to the one-electron reduction of one of the two **BPY**^+•^ units in the **CBPQT**^2(+•)^ ring (**CBPQT**^2(+•)^/**CBPQT**^(+•)^), which is not strongly engaged in hetero radical-paring interactions with the **NDI**^─•^ radical anion of the dumbbell component of **5**^4+^•4PF_6_^─^. The consequence is that this reduction peak does not undergo remarkable shift, compared to the second reduction peak (─0.65 V) of the free **CBPQT**^4+^. In contrast, the fifth reduction peak of the [2]rotaxane **5**^4+^•4PF_6_^─^, which can be attributed to one-electron reduction (**CBPQT**^(+•)^/**CBPQT**), is shifted to a substantially more negative value (─0.73 V). This negative shift can be attributed to the stabilizing influence of the hetero radical-pairing interaction between the **NDI**^─•^ in the dumbbell component of the rotaxane and the **BPY**^+•^ in the **CBPQT**^(+•)^ ring, which renders the reduction of **BPY**^+•^ more difficult. This negative shift also further convinces the encirclement of the **CBPQT**^2(+•)^ or **CBPQT**^(+•)^ around the **NDI**^─•^ unit of the dumbbell component. The sixth reduction peak observed ─0.89 V can be attributed to one-electron reduction (**NDI**^─•^/**NDI**^2─^). This peak does not undergo remarkable shift compared to the second reduction peak of the **NDI**^─•^ unit in the dumbbell compound **8**, an observation indicating that the hetero radical pairing was jeopardized after the ring is fully reduced to its neutral state, namely **CBPQT**, which no longer encircles the **NDI**^─•^ anion.

## Discussion

π–π radical spin–spin pairing interactions between two identical π-electron radical compounds have been studied extensively in the field of supramolecular chemistry. However, the π–π interactions between two different π-electron radicals are seldom employed in driving supramolecular recognition. The lack of hetero radical spin–spin interactions might be explained by the often-occurring mismatch of the SOMOs of two different radical moieties. Here, we demonstrate that with the assistance of a covalently connected system, a bipyridinium radical cation and a naphthalene diimide radical anion can undergo spin–spin interactions. This previously unreported supramolecular recogntion, takes advantage of the marriage of hetero radical spin–spin pairing interactions and Coulombic attraction. Theoretical calculation demonstrates that the latter interaction might play a more predominant role, especially in the case when these two radical moieties orientate in a center-aligned manner. We employed this noncovalent interaction to drive the formation of a pseudorotaxane, which is composed of a cyclophane host containing two bipyridinium radical cations encircling a naphthalene diimide radical anionic guest. This recognition motif can also be harnessed to drive the intramolecular mechanical movement of a bistable [2]rotaxane that can function as a redox-stimulated molecular switch. The discovery of this nocovalent interaction opens up opportunities for rationally designing supramolecular or mechanically interlocked architectures with more diverse functions.

## Methods

### Synthesis

The syntheses of model compounds **2**, **3**^2+^•2PF_6_^─^, and **4**, as well as azide and alkyne derivatives, namely **6** and **7**, were described in Supplementary Figs. 1–12. The dumbbell **8** and the [2]rotaxane **5**^4+^•4PF_6_^−^ were synthesized by using a copper(I)-catalyzed alkyne-azide cyclization.

### Compound characterization

NMR spectra were recorded at ambient temperature using Bruker AVANCE III 400/500 spectrometers, with working frequencies of 400/500 and 100/125 MHz for ^1^H and ^13^C, respectively. Chemical shifts are reported in ppm relative to the residual internal non-deuterated solvent signals (CD_3_CN: *δ* = 1.94 ppm; CDCl_3_: *δ* = 7.26 ppm). High-resolution mass spectra were recorded on an Fourier transform ion cyclotron resonance mass spectrometry. EPR spectra were taken on a cw-EPR spectrometer (Bruker A300). CV experiments was carried out at room temperature in argon-purged solutions in MeCN with a Gamry Multipurpose instrument (Reference 600) interfaced to a PC. UV/Vis/NIR absorption spectra were taken on a Cary Series UV-Vis-NIR spectrophotometer.

### Data availability

The authors declare that all other data supporting the findings of this study are available from the article and its Supplementary Information files or available from the authors upon reasonable request.

## Electronic supplementary material

Supplementary Information
