# Supplementary material for: Coulombic-enhanced hetero radical pairing interactions
Source: Nat Commun. 2018 May 17;9:1961. doi: 10.1038/s41467-018-04335-0 (PMC5958055; doi:10.1038/s41467-018-04335-0)
Supplement: Supplementary file 1 — Supplementary Information [file 41467_2018_4335_MOESM1_ESM.pdf]

Coulombic-Enhanced Hetero Radical Pairing Interactions

Xujun Zheng[1,2†], Yang Zhang[1†], Ning Cao[1†], Xin Li[1], Shuoqing Zhang[1], Renfeng Du[1], Haiying Wang[3], Zhenni Ye[1], Yan Wang[1], Fahe Cao[1], Haoran Li[1], Xin Hong[1], Andrew C.-H. Sue[3], Chuluo Yang[2], Wei-Guang Liu[4] and Hao Li[1*]

[1] Department of Chemistry, Zhejiang University, Hangzhou 310027, P. R. China. [2] Hubei Collaborative Innovation Center for Advanced Organic Chemical Materials, Hubei Key Lab on Organic and Polymeric Optoelectronic Materials, Department of Chemistry, Wuhan University, Wuhan 430072, P. R. China. [3] Institute for Molecular Design and Synthesis, School of Pharmaceutical Science & Technology, Health Science Platform, Tianjin University, 92 Weijin Road, Nankai District, Tianjin 300072, People's Republic of China. [4]Department of Chemistry, Chemical Theory Center, and Minnesota Supercomputing Institute, University of Minnesota, Minneapolis, Minnesota 55455-0431, United States.

[†]These authors contributed equally to this work. [*]e-mail: lihao2015@zju.edu.cn

Supporting Information

1. General Methods

All reagents and solvents were purchased from commercial sources and used without further purification. All experiments involving radicals were handled in glove boxes. Cyclobis(paraquat-p-phenylene) tetrakis(hexafluorophosphate) (**CBPQT**$^{4+}$•4PF_6^-),[1] compound **S4**[2] and **S9**[3] were prepared according to literature procedures. Nuclear magnetic resonance (NMR) spectra were recorded at ambient temperature using Bruker AVANCE III 400/500 spectrometers, with working frequencies of 400/500 and 100/125 MHz for ^{1}H and ^{13}C, respectively. Chemical shifts are reported in ppm relative to the residual internal non-deuterated solvent signals (CD_3CN: δ = 1.94 ppm; $CDCl_3$: δ = 7.26 ppm). High-resolution mass spectra (HRMS) were recorded on an Fourier transform ion cyclotron resonance mass spectrometry (FT-ICR MS). Cyclic voltammetry experiments (CV) was carried out at room temperature in argon-purged solutions in MeCN with a Gamry Multipurpose instrument (Reference 600) interfaced to a PC. CV experiments were performed using a glassy carbon working electrode (0.018 cm^2, Cypress system). The electrode surface was polished routinely with a 0.05 μm alumina-water slurry on a felt surface immediately before use. The counter electrode was a Pt coil and the reference electrode was a Ag/AgCl electrode. The concentration of the sample and supporting electrolyte tetrabutylammonium hexafluorophosphate ($TBAPF_6$) were 1.0 × 10^{-3} mol L^{-1} and 0.1 mol L^{-1}, respectively. The scan rate was set to 200 mV s^{-1}. Electron paramagnetic resonance (EPR) spectra were taken on a cw-EPR spectrometer (Bruker A300), and the samples were injected into a glass capillary and put into the cavity. The EPR spectra were recorded in X-band at room temperature with a microwave power of 0.2 mW, a modulation frequency of 100 kHz and modulation amplitude to 1G. The g-factors were corrected with respect to that of an equipped g-marker (DPPH, g = 2.0036). UV/Vis/NIR absorption spectra were taken on a Cary Series UV-Vis-NIR spectrophotometer.

2. Synthetic Procedures

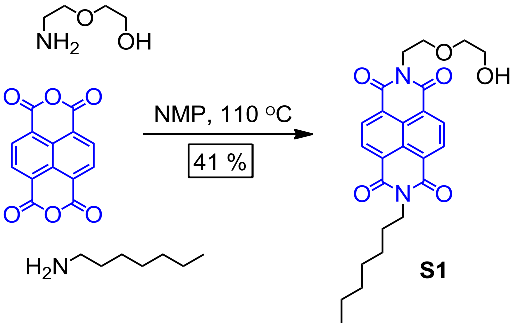

Supplementary Figure 1. Synthesis of **S1**

S1: To a stirred solution of naphthalene-1,4,5,8-tetracarboxylic dianhydride (1.34 g, 5.0 mmol) in 1-methyl-2-pyrrolidone (NMP) (50 mL), n-heptylamine (0.74 mL, 5.0 mmol) and 2-(2-aminoethoxy)ethanol (0.50 mL, 5.0 mmol) was added. The mixture was stirred at 110 ℃ for 2 hours, then poured into water and the pink solid was collected by filtration. The crude product was purified by silica gel chromatography (eluent: CH_2Cl_2/MeOH = 20/1, v/v) to yield **S1** (0.93 g, 41%) as a light pink solid. ^{1}H NMR (400 MHz, $CDCl_3$) δ 8.77 (s, 4H), 4.48 (t, J = 5.6 Hz, 2H), 4.23 – 4.15 (m, 2H), 3.88 (t, J = 5.6 Hz, 2H), 3.75 – 3.62 (m, 4H), 2.22 (t, J = 5.6 Hz, 1H), 1.73 (m, 2H), 1.48 – 1.23 (m, 8H), 0.92 – 0.84 (m, 3H). ^{13}C NMR (100 MHz, $CDCl_3$) δ 163.2, 162.8, 131.2, 130.9, 126.8, 126.4, 72.2, 68.2, 61.8, 41.0, 40.0, 31.7, 29.0, 28.1, 27.0, 22.6, 14.1. HRMS (ESI): *m/z* Calcd for $C_{25}H_{28}N_2NaO_6$: 475.1845, Found: 475.1848 (M + Na)$^+$.

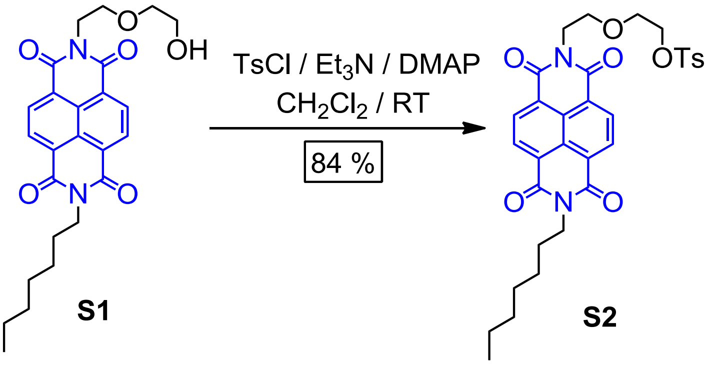

Supplementary Figure 2. Synthesis of **S2**

S2: Compound **S1** (904 mg, 2.0 mmol), DMAP (6.4 mg, 0.5 mmol), and triethylamine

(16 ml) were stirred in dichloromethane (100 ml) at 0 ℃ for 1 hour. *p*-Toluenesulfonyl chloride (475 mg, 2.5 mmol) in dichloromethane (20 ml) was added drop-wise and the mixture was stirred at room temperature for 8 h. Water was added to the reaction mixture and the compound was extracted using DCM and chromatographed on silica (eluent: CH_2Cl_2) to yield **S2** as a pink solid (1018 mg, 84%). ^{1}H NMR (400 MHz, $CDCl_3$) δ 8.75 (s, 4H), 7.71 (d, *J* = 8.4 Hz, 2H), 7.30 (d, *J* = 8.4 Hz, 2H), 4.41 (t, *J* = 5.6 Hz, 2H), 4.22 – 4.16 (m, 2H), 4.13 – 4.08 (m, 2H), 3.80 (t, *J* = 5.6 Hz, 2H), 3.74 – 3.69 (m, 2H), 2.42 (s, 3H), 1.74 (m, 2H), 1.45 – 1.24 (m, 8H), 0.90 – 0.85 (m, 3H). ^{13}C NMR (100 MHz, $CDCl_3$) δ 163.0, 162.8, 131.1, 130.9, 129.8, 127.9, 126.7, 126.4, 69.1, 68.2, 67.9, 41.0, 39.4, 31.7, 29.0, 28.1, 27.1, 22.6, 21.7, 14.1. HRMS (ESI): *m/z* Calcd for $C_{32}H_{34}N_2NaO_8S$: 629.1934, Found: 629.1924 $(M + Na)^+$.

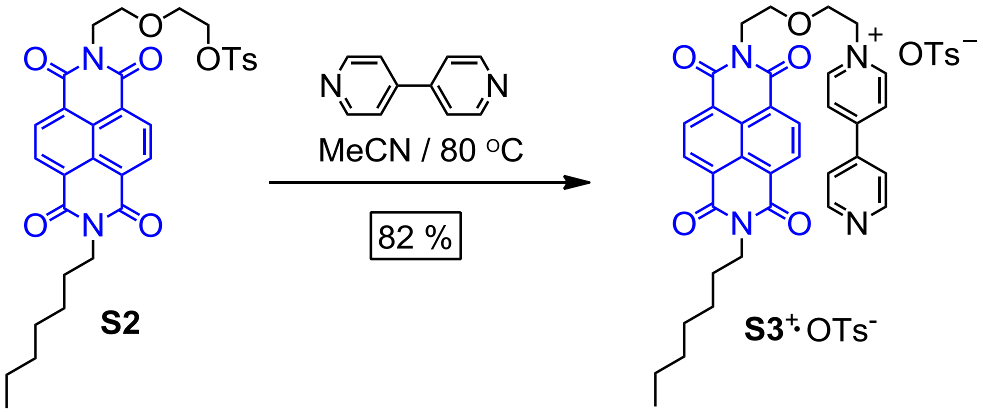

Supplementary Figure 3. Synthesis of **S3**$^+$•OTs$^−$

S3$^+$•OTs$^−$: Compound **S2** (909 mg, 1.5 mmol) and 4,4′-bipyridine (936 mg, 6.0 mmol) were dissolved in MeCN (20 mL). The mixture was stirred at 80 ℃ for 2 days. After cooling to room temperature, the pale yellow solid was collected by filtration, and washed with ethyl ether to give the desired product **S3**$^+$•OTs$^−$ (937 mg, 82%). ^{1}H NMR (400 MHz, $CDCl_3$) δ 9.36 (d, *J* = 6.8 Hz, 2H), 8.89 – 8.84 (m, 2H), 8.70 (s, 4H), 8.16 (d, *J* = 6.8 Hz, 2H), 7.73 – 7.67 (m, 2H), 7.60 – 7.54 (m, 2H), 7.10 (d, *J* = 7.6 Hz, 2H), 5.06 (t, *J* = 4.8 Hz, 2H), 4.38 (t, *J* = 4.8 Hz, 2H), 4.24 – 4.15 (m, 2H), 4.02 (t, *J* = 4.8 Hz, 2H), 3.81 (t, *J* = 4.8 Hz, 2H), 2.30 (s, 3H), 1.74 (m, 2H), 1.49 – 1.23 (m, 8H), 0.92 – 0.84 (m, 3H). ^{13}C NMR (100 MHz, $CDCl_3$) δ 163.0, 162.5, 153.4, 151.4, 146.8,

143.6, 141.0, 139.3, 131.0, 130.8, 128.6, 126.8, 126.7, 126.6, 126.2, 125.8, 124.9, 121.3, 68.7, 68.5, 61.3, 41.0, 39.9, 31.7, 29.0, 28.1, 27.0, 22.6, 21.3, 14.1. HRMS (ESI): *m/z* Calcd for $C_{35}H_{35}N_4O_5$: 591.2602, Found: 591.2597 (M - OTs)$^+$.

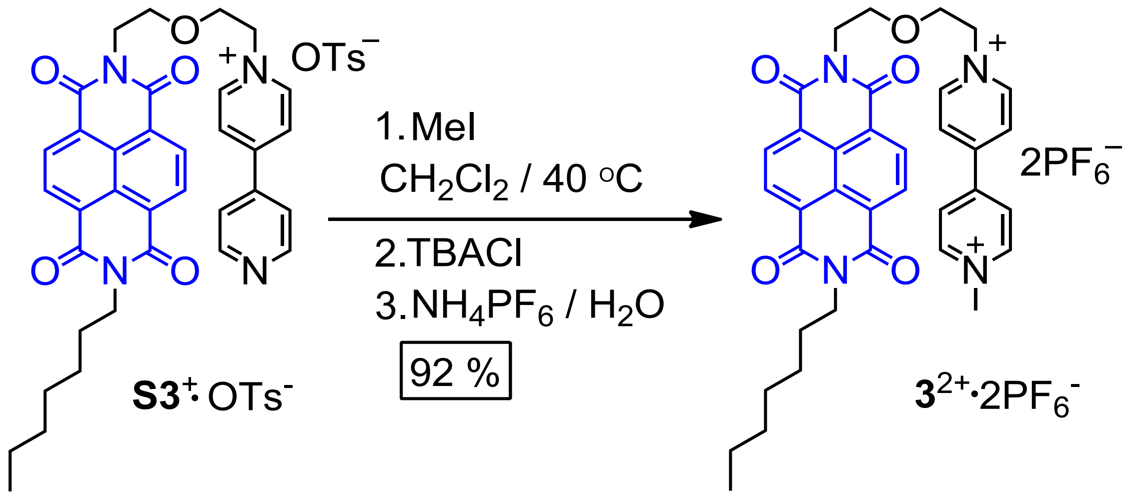

Supplementary Figure 4. Synthesis of $\mathbf{3}^{2+}$•$2PF_6^-$

$\mathbf{3}^{2+}$•$2PF_6^-$: Compound **S3**$^+$•OTs^- (762 mg, 1.0 mmol) and iodomethane (568 mg, 4.0 mmol) were dissolved in dry CH_2Cl_2 (20 mL). The reaction mixture was stirred at 40 ℃ for 3 days. The solvent was evaporated, and the residue was dissolved into MeCN (100 mL), tetrabutylammonium chloride (TBA^+•Cl^-, 1g) was then added to the solution. The white precipitate was collected and washed with MeCN to give the desired folding molecules $\mathbf{3}^{2+}$•$2Cl^-$ (622 mg, 92%). Then the solid was dissolved in H_2O (20 mL), followed by adding NH_4PF_6 (2 g). The white produce was collected and washed with ethyl ether to give $\mathbf{3}^{2+}$•$2PF_6^-$ (0.89 g, 99%). ^{1}H NMR (400 MHz, CD_3CN) δ 8.81 (dd, *J* = 6.8, 3.4 Hz, 4H), 8.56 – 8.49 (dd, *J* = 7.6, 1.2 Hz, 2H), 8.45 (dd, *J* = 7.6, 1.2 Hz, 2H), 8.23 (d, *J* = 6.4 Hz, 2H), 8.17 (d, *J* = 6.4 Hz, 2H), 4.78 – 4.71 (m, 2H), 4.46 (s, 3H), 4.28 (t, *J* = 5.2 Hz, 2H), 4.16 – 4.09 (m, 2H), 3.97 (t, *J* = 5.2 Hz, 2H), 3.82 (t, *J* = 5.2 Hz, 2H), 1.71 (m, 2H), 1.48 – 1.25 (m, 8H), 0.94 – 0.86 (m, 3H). ^{13}C NMR (100 MHz, CD_3CN) δ 162.7, 162.6, 149.0, 146.1, 146.0, 130.0, 129.8, 126.5, 126.3, 126.1, 126.0, 67.8, 67.2, 61.4, 48.4, 40.3, 39.0, 31.2, 28.4, 27.3, 26.5, 22.0, 13.1. HRMS (ESI): *m/z* Calcd for $C_{36}H_{38}N_4O_5$: 303.1416, Found: 303.1409 (M – $2PF_6$)$^{2+}$. *m/z* Calcd for $C_{36}H_{38}F_6N_4O_5P$: 751.2473, Found: 751.2481 (M – PF_6)$^+$.

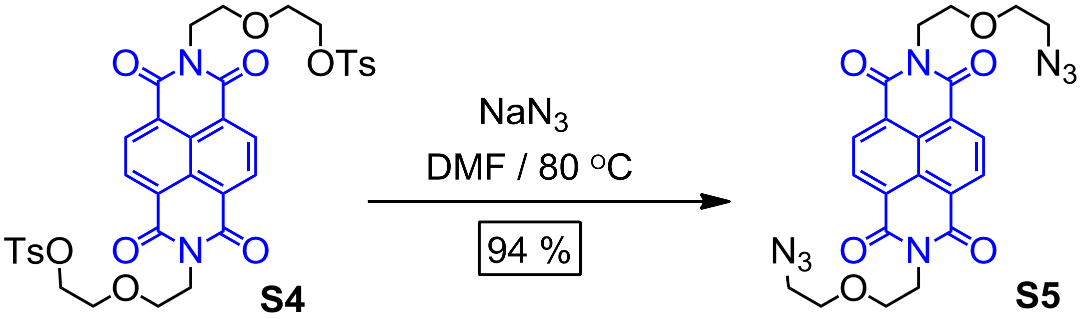

Supplementary Figure 5. Synthesis of **S5**

S5: Compound **S4** (375 mg, 0.5 mmol) and NaN_3 (98 mg, 1.5 mmol) were dissolved in DMF (20 mL). The reaction mixture was stirred at 80 ℃ for 16 h. After cooling down to room temperature, the solution was poured into H_2O (50 mL). The resulting mixture was extracted with EtOAc (3 x 20 mL) and the combined organic phases were washed three times with saturated aqueous NaCl solution (3 x 100 mL). After dried by using $MgSO_4$, the solvent was removed in vacuo to afford the desired product **S5** (231 mg, 94%) as a white solid, which was used immediately in the next step without further purification. ^{1}H NMR (400 MHz, CD_3CN) δ 8.68 (s, 4H), 4.36 (t, *J* = 6.0 Hz, 4H), 3.80 (t, *J* = 6.0 Hz, 4H), 3.67 (t, *J* = 4.8 Hz, 4H), 3.30 (t, *J* = 4.8 Hz, 4H). ^{13}C NMR (100 MHz, $CDCl_3$) δ 163.0, 131.0, 126.8, 126.6, 69.8, 67.9, 50.7, 39.6. HRMS (ESI): *m/z* Calcd for $C_{22}H_{20}N_8NaO_6$: 515.1404, Found: 515.1397 $(M + Na)^+$.

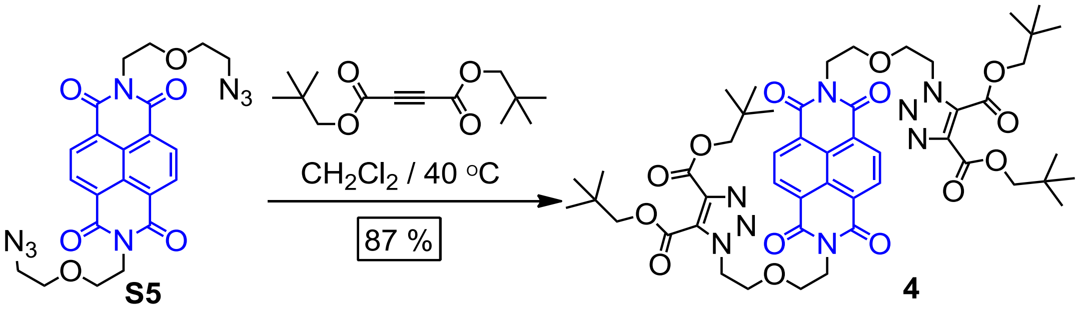

Supplementary Figure 6. Synthesis of the dumbbell **4**

4: Compound **S5** (123 mg, 0.25 mmol) and di-*tert*-butyl acetylenedicarboxylate (254 mg, 1 mmol) were dissolved in CH_2Cl_2 (10 mL). The mixture was stirred at 40 ℃ for 48 h. The solvent was evaporated and the crude product was purified by silica gel chromatography (eluent: EtOAc) to yield **4** (218 mg, 87%) as an orange solid. ^{1}H NMR (400 MHz, $CDCl_3$) δ 8.71 (s, 4H), 4.78 (t, *J* = 5.2 Hz, 4H), 4.37 (t, *J* = 5.6 Hz,

4H), 3.97 (m, 8H), 3.92 (s, 4H), 3.79 (t, J = 5.6 Hz, 4H), 0.94 (s, 18H), 0.92 (s, 18H). ^{13}C NMR (100 MHz, $CDCl_3$) δ 162.9, 160.2, 158.7, 139.9, 131.1, 130.7, 126.8, 126.4, 75.9, 74.8, 68.3, 67.7, 50.0, 38.9, 31.5, 31.3, 26.4, 26.3. HRMS (ESI): m/z Calcd for $C_{50}H_{64}N_8NaO_{14}$: 1023.4440, Found: 1023.4445 $(M + Na)^+$.

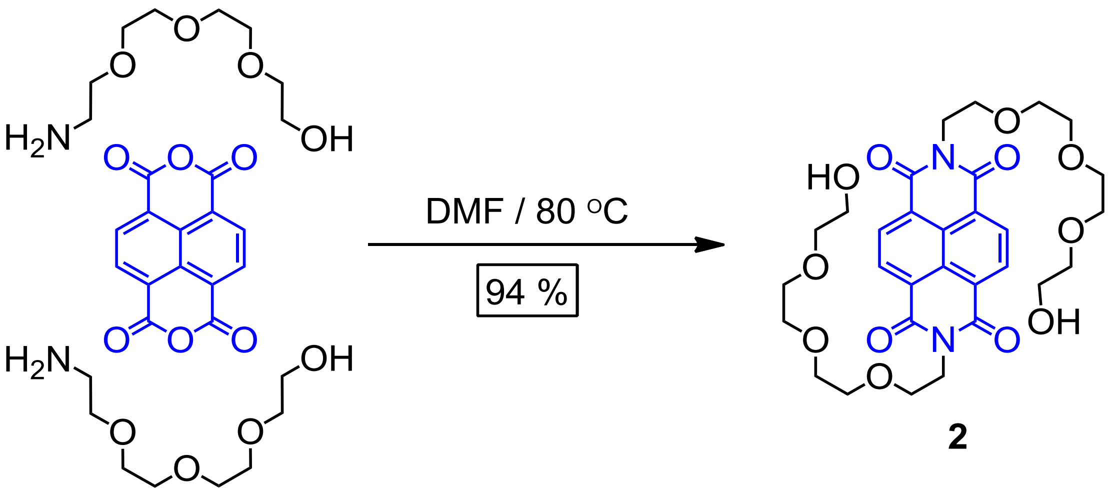

Supplementary Figure 7. Synthesis of the **NDI** derivative **2**

2: To a stirred solution of naphthalene-1,4,5,8-tetracarboxylic dianhydride (268 mg, 1.0 mmol) in DMF (20 mL), 2-(2-(2-(aminomethoxy)ethoxy)ethoxy)ethanol (0.36 ml, 2.0 mmol) was added. The mixture was stirred at 80 ℃ for 6 hours, and then poured into water. The mixture was extracted with CH_2Cl_2 (3 × 20 mL). The organic layer was washed with brine and dried over anhydrous $MgSO_4$. After concentrated in vacuum, the dark red crude product was obtained and purified by column chromatography (eluent: CH_2Cl_2/MeOH = 15/1, v/v), yielding **2** (581 mg, 94%) as a yellow solid. ^{1}H NMR (400 MHz, $CDCl_3$) δ 8.76 (s, 4H), 4.47 (t, J = 5.6 Hz, 4H), 3.86 (t, J = 5.6 Hz, 4H), 3.73 – 3.69 (m, 4H), 3.65 – 3.60 (m, 8H), 3.60 – 3.55 (m, 8H), 3.51 (m, 4H), 3.48 (s, 2H). ^{13}C NMR (100 MHz, $CDCl_3$) δ 162.9, 130.9, 126.7, 126.6, 72.5, 70.5, 70.5, 70.2, 70.1, 67.8, 61.6, 39.6. HRMS (ESI): m/z Calcd for $C_{30}H_{38}N_2NaO_{12}$: 641.2322, Found: 641.2326 $(M + Na)^+$.

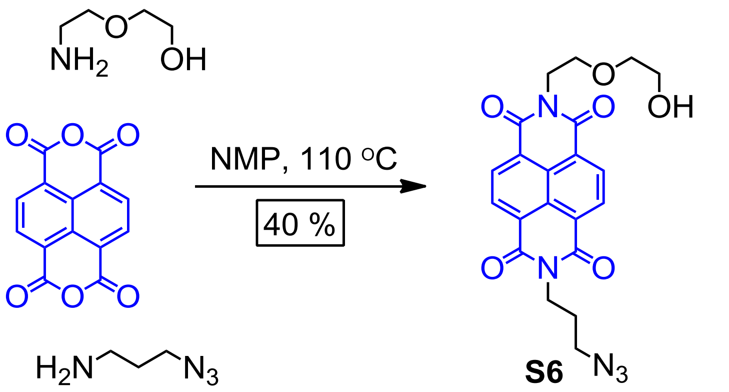

Supplementary Figure 8. Synthesis of **S6**

S6: To a stirred solution of naphthalene-1,4,5,8-tetracarboxylic dianhydride (1.34 g, 5.0 mmol) in 1-methyl-2-pyrrolidone (NMP) (50 mL), 3-azidopropan-1-amine (0.5 g, 5.0 mmol) and 2-(2-aminoethoxy)ethanol (0.50 mL, 5.0 mmol) was added. The mixture was stirred at 110 ℃ for 6 hours, then poured into water. The light yellow precipitate were collected by filtration. The crude product was purified by silica gel chromatography (eluent: CH_2Cl_2/MeOH = 20/1, v/v) to yield **S6** (874 mg, 40%) as a light yellow solid. ^{1}H NMR (400 MHz, $CDCl_3$). δ 8.76 (m, 4H), 4.47 (t, *J* = 5.6 Hz, 2H), 4.31 (t, *J* = 7.2 Hz, 2H), 3.87 (t, *J* = 5.6 Hz, 2H), 3.71 – 3.63 (m, 4H), 3.46 (t, *J* = 6.8 Hz, 2H), 2.27 (s, 1H), 2.05 (m, 2H). ^{13}C NMR (100 MHz, $CDCl_3$) δ 163.1, 162.8, 131.2, 131.1, 126.8, 126.7, 126.6, 126.5, 72.3, 68.2, 61.8, 49.4, 40.0, 38.5, 27.5. HRMS (ESI): *m/z* Calcd for $C_{21}H_{19}N_5NaO_6$: 460.1233, Found: 460.1232 $(M + Na)^+$.

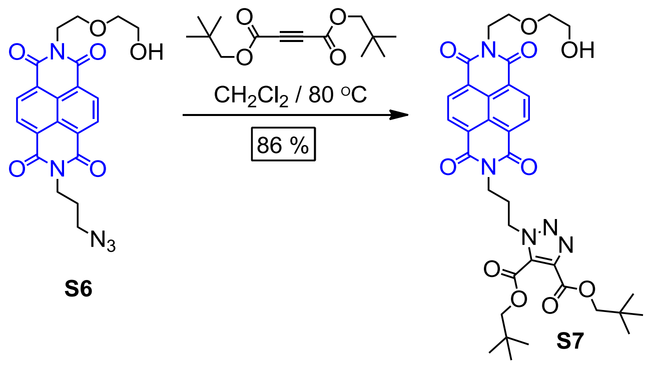

Supplementary Figure 9. Synthesis of **S7**

S7: Compound **S6** (437 mg, 1 mmol) and di-*tert*-butyl acetylenedicarboxylate (508 mg, 2 mmol) were dissolved in CH_2Cl_2 (10 mL). The mixture was stirred at 40 ℃ for

48 h. The solvent was evaporated and the crude product was purified by silica gel chromatography (eluent: CH_2Cl_2/MeOH = 20/1, v/v) to yield **S7** (594 mg, 86%) as a yellow solid. ^{1}H NMR (500 MHz, CD_3CN) δ 8.58 (d, *J* = 7.5 Hz, 2H), 8.53 (d, *J* = 7.5 Hz, 2H), 4.69 (t, *J* = 7.0 Hz, 2H), 4.32 – 4.28 (m, 2H), 4.19 (t, *J* = 7.0 Hz, 2H), 4.02 (s, 2H), 4.00 (s, 2H), 3.75 (m, 2H), 3.55 (m, 4H), 2.73 (t, *J* = 5.5 Hz, 1H), 2.39 (m, 2H), 0.96 (s, 9H), 0.94 (s, 9H). ^{13}C NMR (100 MHz, $CDCl_3$) δ 163.0, 162.8, 160.4, 158.6, 131.1, 131.1, 129.2, 126.6, 126.5, 126.4, 76.1, 74.9, 72.3, 68.1, 61.7, 48.6, 40.0, 38.0, 31.6, 31.4, 28.6, 26.4, 26.4. HRMS (ESI): *m/z* Calcd for $C_{35}H_{41}N_5NaO_{10}$: 714.2751, Found: 714.2747 $(M + Na)^+$.

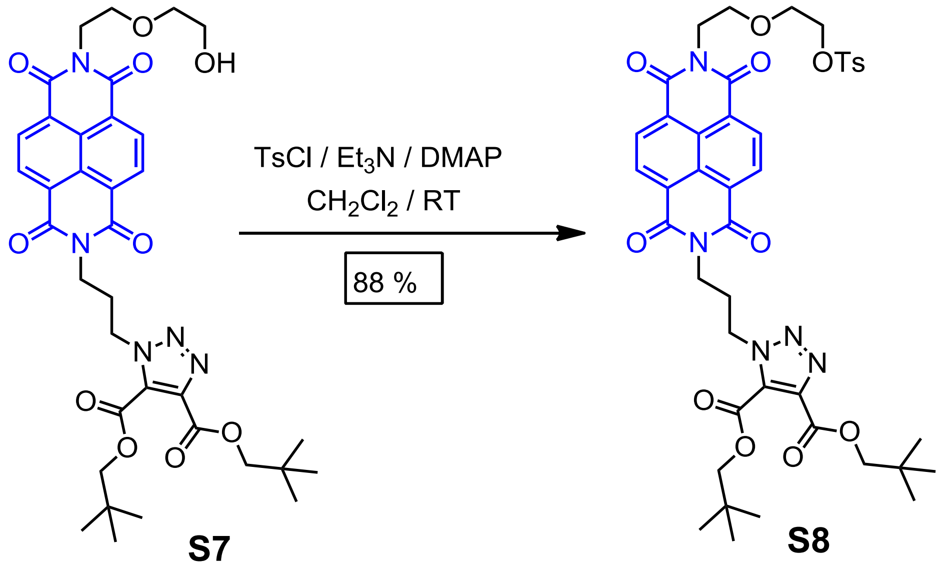

Supplementary Figure 10. Synthesis of **S8**

S8: Compound **S7** (553 mg, 0.8 mmol), 4-dimethylaminopyridine (DMAP, 6.4 mg, 0.5 mmol), and triethylamine (10 ml) were stirred in dichloromethane (20 ml) at 0 ℃ for 1 hour. *p*-Toluenesulfonyl chloride (228 mg, 1.2 mmol) in dichloromethane (10 ml) was added and the mixture was stirred at room temperature for 8 h. Water was added to the reaction mixture and the compound was extracted using DCM. After removing the solvent under reduced pressure, a crude product was obtained and then purified by silica gel chromatography (eluent: CH_2Cl_2), yielding **S8** as a light green waxy solid (595 mg, 88%). ^{1}H NMR (400 MHz, $CDCl_3$) δ 8.75 (m, 4H), 7.71 (d, *J* = 8.0 Hz, 2H), 7.30 (d, *J* = 8.0 Hz, 2H), 4.76 (t, *J* = 7.6 Hz, 2H), 4.42 (t, *J* = 5.6 Hz, 2H), 4.35 (t, *J* =

6.8 Hz, 2H), 4.17 – 4.09 (m, 2H), 4.06 (s, 2H), 4.00 (s, 2H), 3.80 (t, *J* = 5.6 Hz, 2H), 3.72 (m, 2H), 2.42 (m, 5H), 1.00 (s, 9H), 0.97 (s, 9H). ^{13}C NMR (100 MHz, $CDCl_3$) δ 162.9, 162.9, 160.5, 132.9, 131.1, 129.8, 129.2, 127.9, 126.7, 126.4, 75.0, 69.2, 68.2, 67.9, 48.6, 39.4, 38.0, 31.6, 31.5, 28.7, 26.5, 26.4, 21.6. HRMS (ESI): *m/z* Calcd for $C_{42}H_{47}N_5NaO_{12}S$: 868.2840, Found: 868.2845 $(M + Na)^+$.

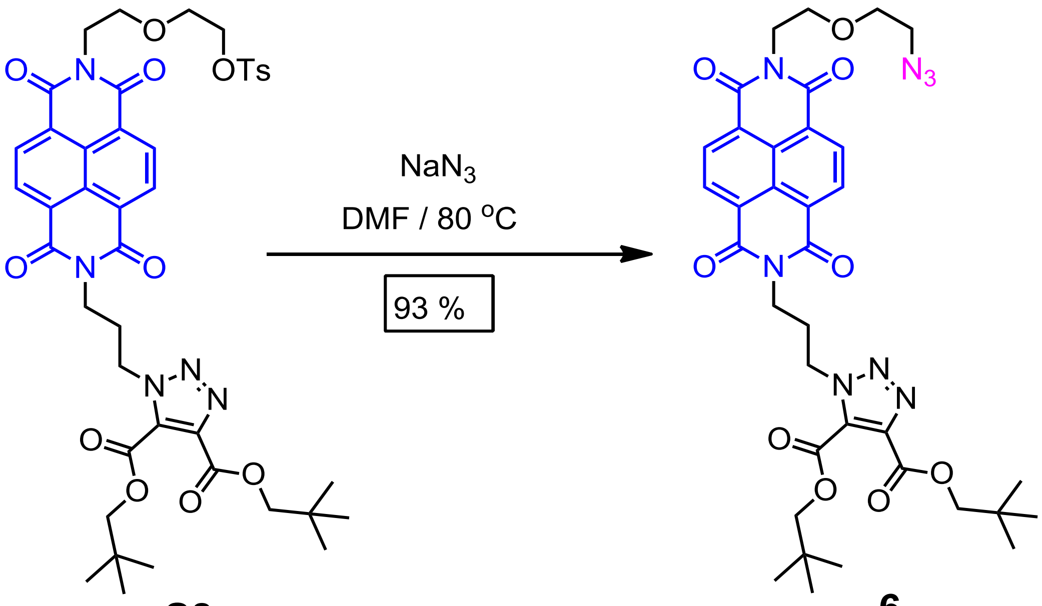

Supplementary Figure 11. Synthesis of **6**

6: Compound **S8** (423 mg, 0.5 mmol) and NaN_3 (49 mg, 0.75 mmol) were dissolved in DMF (20 mL). The reaction mixture was stirred at 80 ℃ for 16 h. After cooling down to room temperature, the solution was poured into H_2O (200 mL). The resulting mixture was extracted with EtOAc (3 x 20 mL) and the crude product was purified by silica gel chromatography (eluent: CH_2Cl_2) to yield **6** (333 mg, 93%) as a yellow waxy solid. ^1H NMR (400 MHz, $CDCl_3$) δ 8.77 (d, *J* = 7.6 Hz, 2H), 8.74 (d, *J* = 7.6 Hz, 2H), 4.76 (t, *J* = 7.6 Hz, 2H), 4.50 (t, *J* = 5.6 Hz, 2H), 4.35 (t, *J* = 6.8 Hz, 2H), 4.06 (s, 2H), 4.00 (s, 2H), 3.89 (t, *J* = 5.6 Hz, 2H), 3.72 (t, *J* = 4.8 Hz, 2H), 3.32 (t, *J* = 4.8 Hz, 2H), 2.44 (m, 2H), 1.00 (s, 9H), 0.97 (s, 9H). ^{13}C NMR (100 MHz, $CDCl_3$) δ 162.8, 158.6, 131.0, 131.0, 126.6, 126.6, 126.3, 76.1, 74.9, 69.8, 67.8, 50.6, 48.6, 39.6, 38.0, 31.6, 31.4, 28.6, 26.4, 26.3. HRMS (ESI): *m/z* Calcd for $C_{35}H_{40}N_8NaO_9$: 739.2816, Found: 739.2823 $(M + Na)^+$.

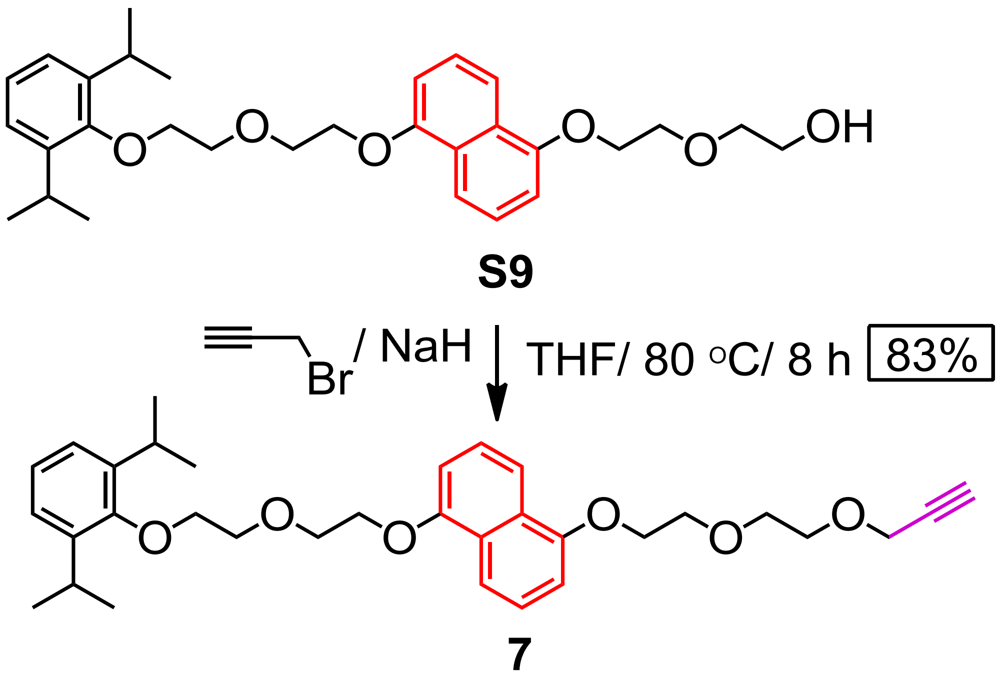

Supplementary Figure 12. Synthesis of **7**

7: Compound **S9** (497 mg, 1.0 mmol), propargyl bromide (357 mg, 3.0 mmol), and NaH (72 mg, 3.0 mmol) were added to a round-bottomed flask (100 mL) containing dry THF (50 mL). The reaction mixture was stirred at 80 ℃ for 8 h. After cooling, the solution was poured into H_2O (100 mL). The resulting mixture was extracted with EtOAc (3 x 20 mL) and the combined organic phases were washed three times with saturated aqueous NaCl solution (3 x 100 mL). After drying ($MgSO_4$), the solvent was removed in vacuo to afford the crude product, which was purified by column chromatography (eluent: Petroleum ether/Ethyl acetate = 20/1, v/v), yielding the desired product **7** (443 mg, 83%) as a light yellow oil. ^{1}H NMR (400 MHz, $CDCl_3$) δ 7.92 (t, *J* = 8.4 Hz, 2H), 7.38 (m, 2H), 7.12 (s, 3H), 6.90 (d, *J* = 7.6 Hz, 1H), 6.86 (d, *J* = 7.6 Hz, 1H), 4.37 (t, *J* = 5.2 Hz, 2H), 4.32 (t, *J* = 4.8 Hz, 2H), 4.24 (d, *J* = 2.4 Hz, 2H), 4.12 (t, *J* = 5.2 Hz, 2H), 4.06 – 3.95 (m, 6H), 3.88 – 3.83 (m, 2H), 3.79 – 3.74 (m, 2H), 3.45 (m, 2H), 2.45 (t, *J* = 2.4 Hz, 1H), 1.24 (d, *J* = 6.8 Hz, 12H). ^{13}C NMR (100 MHz, $CDCl_3$) δ 154.4, 154.3, 141.9, 125.1, 124.7, 124.0, 114.7, 114.7, 105.8, 105.7, 74.7, 74.0, 70.9, 70.8, 70.1, 69.9, 69.2, 68.1, 68.0, 58.5, 26.3, 24.2. HRMS (ESI): *m/z* Calcd for $C_{33}H_{42}NaO_6$: 557.2879, Found: 557.2882 $(M + Na)^+$.

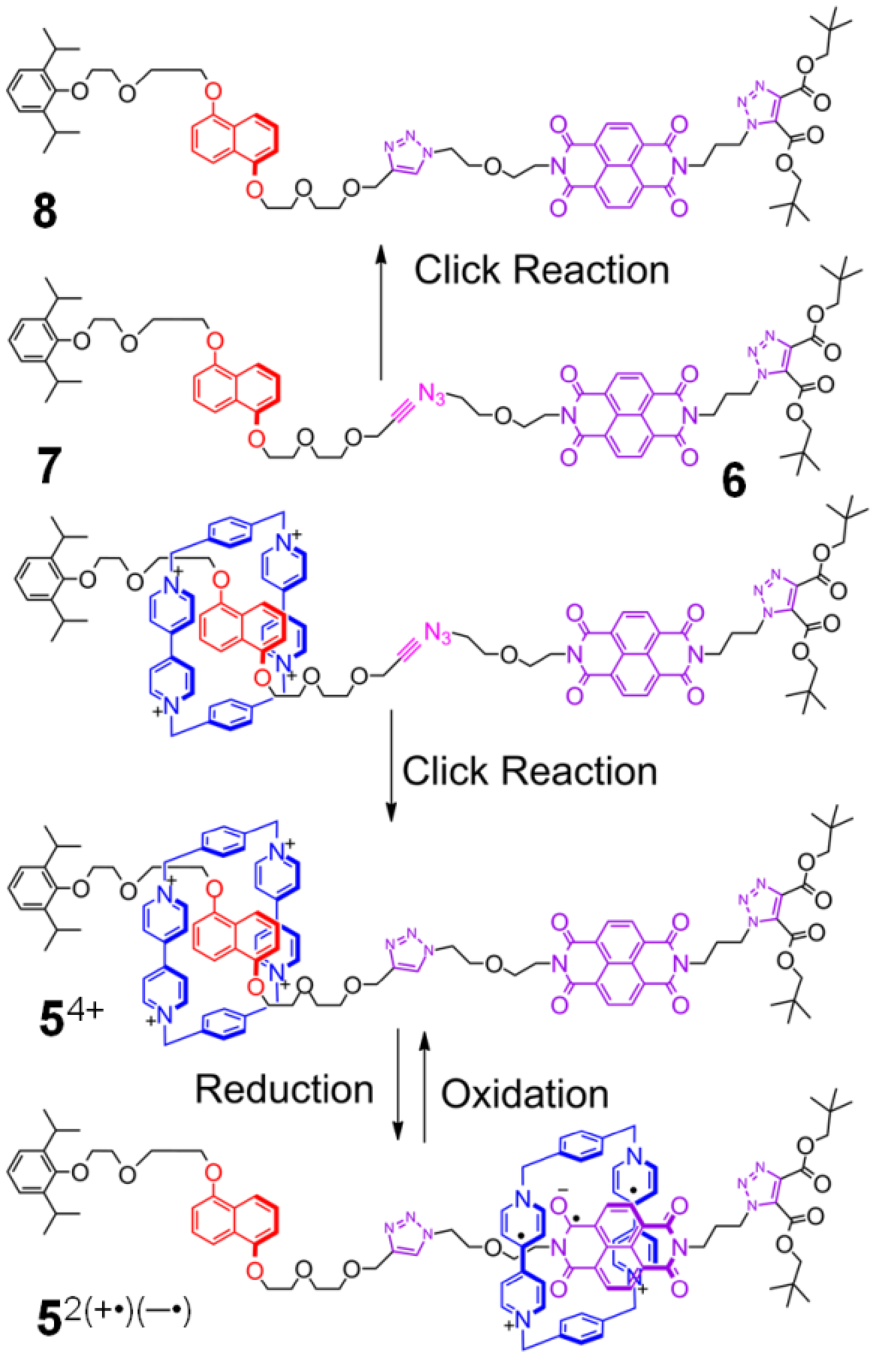

Supplementary Figure 13. Synthesis of dumbbell **8** and the [2]rotaxane $\mathbf{5}^{4+}$•$4PF_6^-$

8: Compound **7** (80 mg, 0.15 mmol), and the azide **6** (129 mg, 0.18 mmol), along with a catalytic amount of $Cu(MeCN)_4PF_6$ and TBTA, were dissolved in Me_2CO (20 mL) at room temperature. The reaction mixture was stirred overnight under argon. The solvent was evaporated and the resulting crude product was purified by column chromatography (eluent: CH_2Cl_2/MeOH = 20/1, v/v) to yield **8** (169 mg, 90%) as a red oil. ^1H NMR (400 MHz, $CDCl_3$) δ 8.65 (d, J = 7.6 Hz, 2H), 8.61 (d, J = 7.6 Hz,

2H), 7.77 (s, 1H), 7.55 (d, *J* = 8.4 Hz, 2H), 7.14 – 7.07 (m, 5H), 6.63 (d, *J* = 7.6 Hz, 2H), 4.77 (t, *J* = 7.2 Hz, 2H), 4.64 (s, 2H), 4.48 – 4.43 (t, *J* = 5.2 Hz, 2H), 4.39 (t, *J* = 5.2 Hz, 2H), 4.32 (t, *J* = 6.8 Hz, 2H), 4.26 – 4.19 (m, 4H), 4.08 – 4.04 (m, 4H), 4.03 – 3.96 (m, 8H), 3.88 – 3.75 (m, 8H), 3.42 (m, 2H), 2.44 (m, 2H), 1.21 (s, 6H), 1.20 (s, 6H), 1.00 (s, 9H), 0.98 (s, 9H). ^{13}C NMR (100 MHz, $CDCl_3$) δ 162.9, 162.8, 160.5, 158.6, 153.9, 153.0, 145.0, 141.9, 140.8, 131.0, 130.9, 129.2, 126.4, 126.3, 126.2, 126.1, 124.8, 124.7, 124.6, 124.0, 123.9, 114.5, 114.4, 105.2, 75.0, 74.0, 71.0, 71.0, 70.1, 70.0, 69.8, 68.9, 68.5, 67.9, 67.8, 64.9, 50.4, 48.7, 39.4, 38.0, 31.6, 31.5, 29.7, 28.7, 26.5, 26.4, 26.3, 24.1. HRMS (ESI): *m/z* Calcd for $C_{68}H_{82}N_8NaO_{15}$: 1273.5797, Found: 1273.5784 $(M + Na)^+$. *m/z* Calcd for $C_{68}H_{82}N_8Na_2O_{15}$: 648.2848, Found: 648.2835 $(M + 2Na)^{2+}$.

5^{4+}•$4PF_6^-$: Compound **7** (80 mg, 0.15 mmol), the azide **6** (129 mg, 0.18 mmol) and **CBPQT**$^{4+}$•$4PF_6^-$ (55 mg, 0.05 mmol), along with a catalytic amount of $Cu(MeCN)_4PF_6$ and TBTA, were dissolved in Me_2CO (20 mL) at room temperature. The reaction mixture was stirred overnight under argon. The solvent was evaporated off and the resulting purple solid was purified by column chromatography [SiO2: 2M NH_4Cl / MeOH / $MeNO_2$ (12 : 7 : 1)]. The solvent was evaporated, followed by adding NH_4PF_6 (2 g) and water (100 mL). The purple solid was collected and washed with water to give **5**$^{4+}$•$4PF_6^-$ (73 mg, 62%). ^{1}H NMR (600 MHz, CD_3CN, 333K) δ 8.84 (s, 8H), 8.70 – 8.64 (m, 4H), 8.04 (s, 8H), 7.66 (s, 1H), 7.42 – 7.28 (m, 8H), 7.15 (m, 3H), 6.37 (d, *J* = 7.8 Hz, 1H), 6.28 (d, *J* = 7.8 Hz, 1H), 6.03 (t, *J* = 8.4 Hz, 1H), 5.99 (t, *J* = 8.4 Hz, 1H), 5.68 (q, *J* = 13.8 Hz, 8H), 4.73 (t, *J* = 7.2 Hz, 2H), 4.49 (s, 2H), 4.44 (s, 2H), 4.37-4.25 (m, 14H), 4.19 (s, 2H), 4.06 – 4.03 (m, 6H), 3.89 (s, 2H), 3.79 (t, *J* = 5.4 Hz, 2H), 3.74 (d, *J* = 6.0 Hz, 2H), 3.47 – 3.41 (m, 2H), 2.56 (d, *J* = 7.8 Hz, 1H), 2.51 (d, *J* = 7.8 Hz, 1H), 2.45 (t, *J* = 7.2 Hz, 2H), 1.14 (s, 6H), 1.13 (s, 6H), 0.99 (s, 18H). HRMS (ESI): *m/z* Calcd for $C_{104}H_{114}N_{12}O_{15}$: 442.7126, Found: 442.7120 $(M – 4PF_6)^{4+}$. *m/z* Calcd for $C_{104}H_{114}F_6N_{12}O_{15}P$: 638.6049, Found: 638.6042 $(M – 3PF_6)^{3+}$. *m/z* Calcd for $C_{104}H_{113}N_{12}O_{15}$: 589.9476, Found: 589.9469 $(M – 4PF_6 – H)^{3+}$. *m/z* Calcd for $C_{104}H_{114}F_{12}N_{12}O_{15}P_2$: 1030.3894, Found: 1030.3899 $(M – 2PF_6)^{2+}$.

3. EPR Analysis

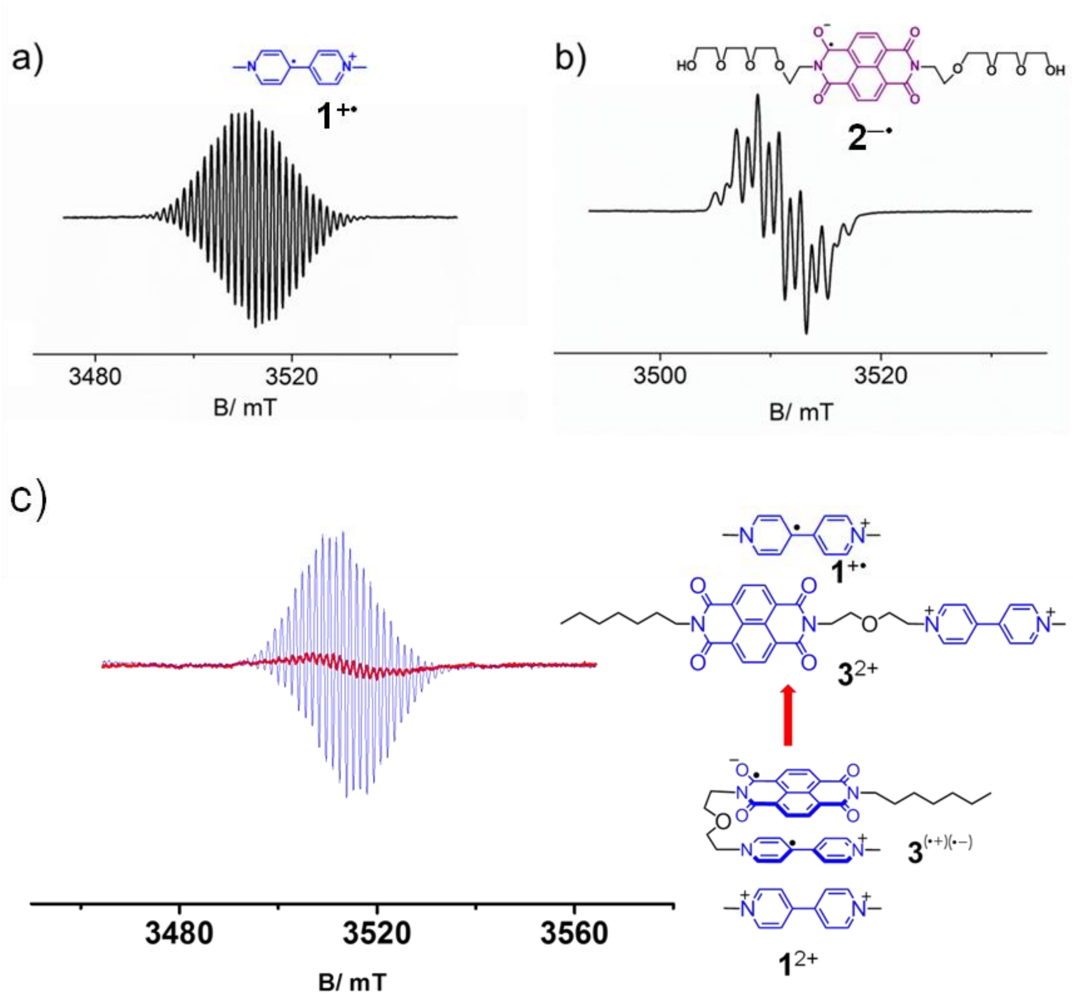

Supplementary Figure 14. EPR spectra of a) **1**$^{+•}$, b) **2**$^{−•}$, as well as c) **3**$^{(+•)(−•)}$ recorded at 25 ^{o}C before (red trace) and after (blue trace) **1**$^{2+}$•$2PF_6^{−}$ (10 mM) was added into the solution. The concentration of **3**$^{(+•)(−•)}$ is 1 mM. The solvent is nitrogen-purged MeCN.

The EPR spectra of **3**$^{(+•)(−•)}$ was recorded (Supplementary Figure 14c, red trace) to justify whether radical pairing occurred. It was observed that the EPR signal of **3**$^{(+•)(−•)}$ was significantly weaker, compared to those of **1**$^{+•}$ and **2**$^{−•}$ with the same concentrations in their individual spectra, an observation indicating that within **3**$^{(+•)(−•)}$, the radical electron in **BPY**$^{+•}$ and **NDI**$^{−•}$ underwent spin paring and became diamagnetic. Addition of **1**$^{2+}$•$2PF_6^{−}$ into the solution of **3**$^{(+•)(−•)}$ produced (Supplementary Figure 14c, bluebox trace) a characteristic spectrum of **1**$^{+•}$ with high

intensity. This observation results from electron transfer form $\mathbf{3}^{(+\bullet)(-\bullet)}$ to $\mathbf{1}^{2+}\bullet 2PF_6^-$, producing $\mathbf{3}^{(+\bullet)}/\mathbf{3}^{2+}$ and $\mathbf{1}^{+\bullet}$, respectively, because $\mathbf{NDI}^{-\bullet}$ is generally more reductive than $\mathbf{BPY}^{+\bullet}$. The EPR spectra of $\mathbf{1}^{+\bullet}$ and $\mathbf{2}^{-\bullet}$ were also recorded (Supplementary Figure 14a, b), which indicate that both of these two species are paramagnetic.

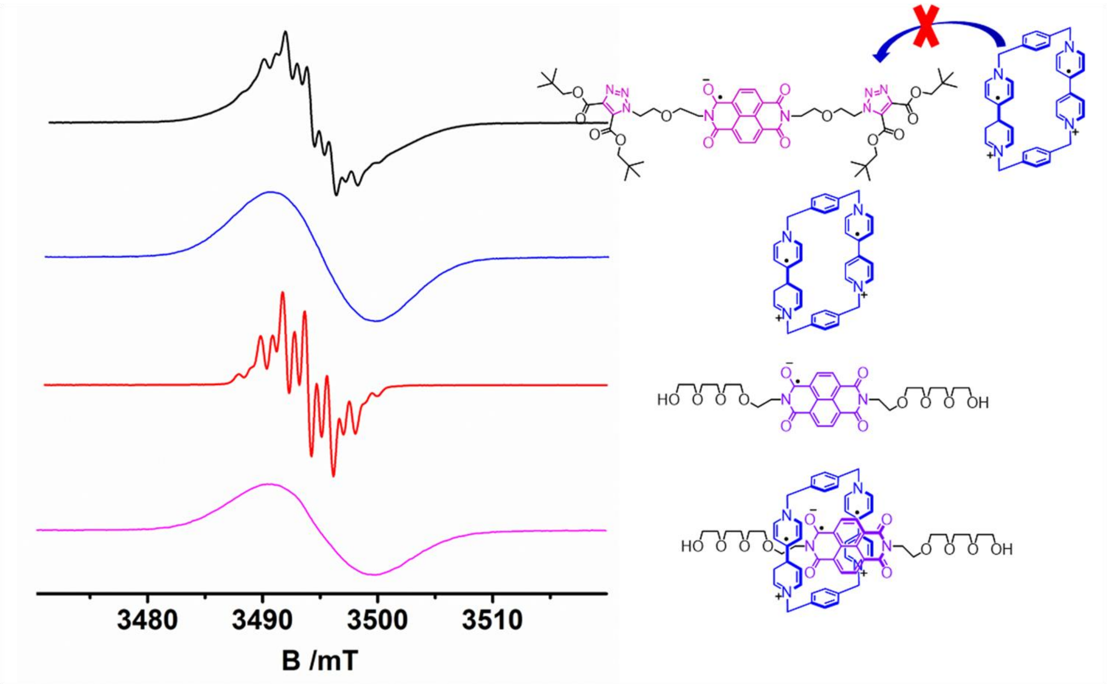

Supplementary Figure 15. EPR spectra of a 1:1 mixture of $\mathbf{4}^{-\bullet}$ and CBPQT$^{2(+\bullet)}$ (black trace, top), CBPQT$^{2(+\bullet)}$ (blue trace), $\mathbf{2}^{-\bullet}$ (red trace), and a 1:1 mixture of $\mathbf{2}^{-\bullet}$ and CBPQT$^{2(+\bullet)}$ (pink trace). The solvent is nitrogen-purged MeCN. The concentrations of all the compounds are 1 mM.

The EPR spectra of a 1:1 mixture of the dumbbell $\mathbf{4}^{-\bullet}$ and $\mathbf{CBPQT}^{2(+\bullet)}$, $\mathbf{CBPQT}^{2(+\bullet)}$, $\mathbf{2}^{-\bullet}$, and a 1:1 mixture of $\mathbf{2}^{-\bullet}$ and $\mathbf{CBPQT}^{2(+\bullet)}$ were also recorded to justify the formation of the pseudorotaxane $\mathbf{NDI}^{-\bullet}\subset\mathbf{CBPQT}^{2(+\bullet)}$. As anticipated, $\mathbf{2}^{-\bullet}$ exhibited hyperfine splitting (Supplementary Figure 15, red trace), consistent with EPR spectra for other **NDI** anionic radicals reported in the literature.[4] The EPR spectrum of $\mathbf{CBPQT}^{2(+\bullet)}$ yielded a total elimination of hyperfine structure (Supplementary Figure 15, blue trace), indicating that the unpaired electrons in $\mathbf{CBPQT}^{2(+\bullet)}$ undergo spin-exchange interactions with each other, which is consistent with the previously reported results.[3] The diradical dicationic $\mathbf{CBPQT}^{2(+\bullet)}$ was then combined with $\mathbf{2}^{-\bullet}$

and the EPR spectrum (Supplementary Figure 15, pink trace) of the mixture was recorded. We observed that the mixture of **CBPQT**$^{2(+\bullet)}$ and **2**$^{-\bullet}$ exhibited no hyperfine structure, indicating that after the formation of the **2**$^{-\bullet}$$\subset$**CBPQT**$^{2(+\bullet)}$ complex, all the unpaired electrons in both **CBPQT**$^{2(+\bullet)}$ and **2**$^{-\bullet}$ underwent spin pairing interaction. In contrast, the EPR spectrum (Supplementary Figure 15, black trace) of the 1:1 mixture of the dumbbell **4**$^{-\bullet}$ and **CBPQT**$^{2(+\bullet)}$ exhibits the superimposition of the spectra of **4**$^{-\bullet}$ and **CBPQT**$^{2(+\bullet)}$ alone, exhibiting hyperfine splitting. This observation indicates that the absence of remarkable interaction between **4**$^{-\bullet}$ and **CBPQT**$^{2(+\bullet)}$, because the bulky terminal stoppers in **4**$^{-\bullet}$ prevent the formation of pseudorotaxane.

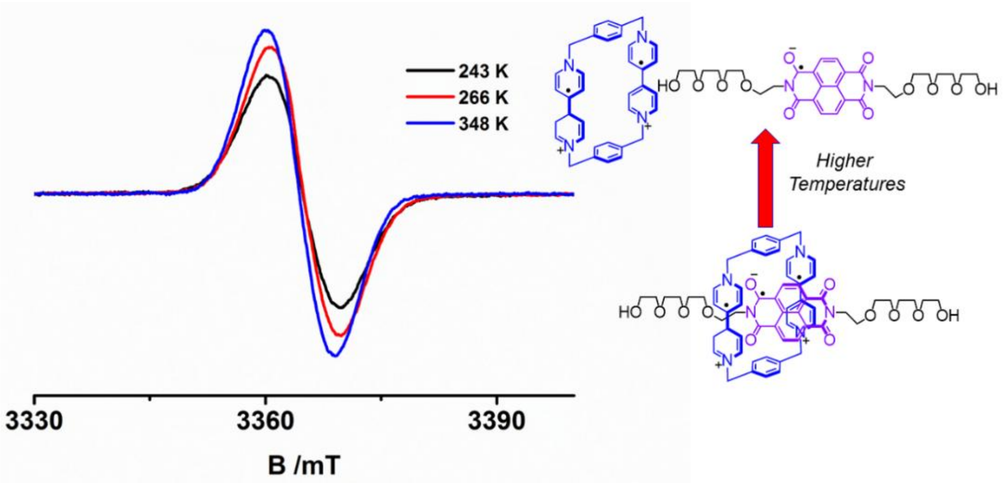

Supplementary Figure 16. EPR spectra of a 1:1 mixture of **2**$^{-\bullet}$ and CBPQT$^{2(+\bullet)}$ at −30 ^{o}C (black trace), −7 ^{o}C (red trace) and 75 ^{o}C (blue trace). The solvent is nitrogen-purged MeCN. The concentrations of all the compounds are 1 mM.

We also recorded the EPR spectra of **2**$^{-\bullet}$$\subset$**CBPQT**$^{2(+\bullet)}$ at variable temperatures (Supplementary Figure 16). We observed that the radical signal of **2**$^{-\bullet}$$\subset$**CBPQT**$^{2(+\bullet)}$ complex was generally larger at higher temperatures (i.e., 75 ^{o}C, Supplementary Figure 16, blue trace) than that at lower temperatures (i.e., −30 ^{o}C, Supplementary Figure 16, black trace). This observation is not surprising, because the formation of **2**$^{-\bullet}$$\subset$**CBPQT**$^{2(+\bullet)}$ complex occurs at the expense of entropy loss. As a consequence, at higher temperature, the complex undergoes dissociation to some extent, increasing its EPR signal. At a lower temperature (−30^{o}C) when the entropy effect is suppressed, more **2**$^{-\bullet}$$\subset$**CBPQT**$^{2(+\bullet)}$ complex forms and the radical signal becomes weaker.

4. Cyclic Voltammetry (CV)

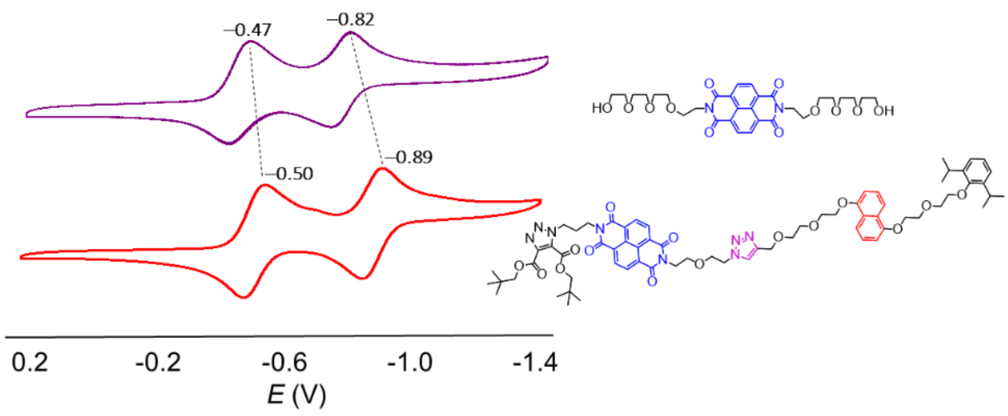

Supplementary Figure 17. Second scans of the cyclic voltammograms (CVs) for **NDI** derivative **2** (purple trace, top), the dumbbell **8** (red trace, bottom)

5. UV/Vis absorption

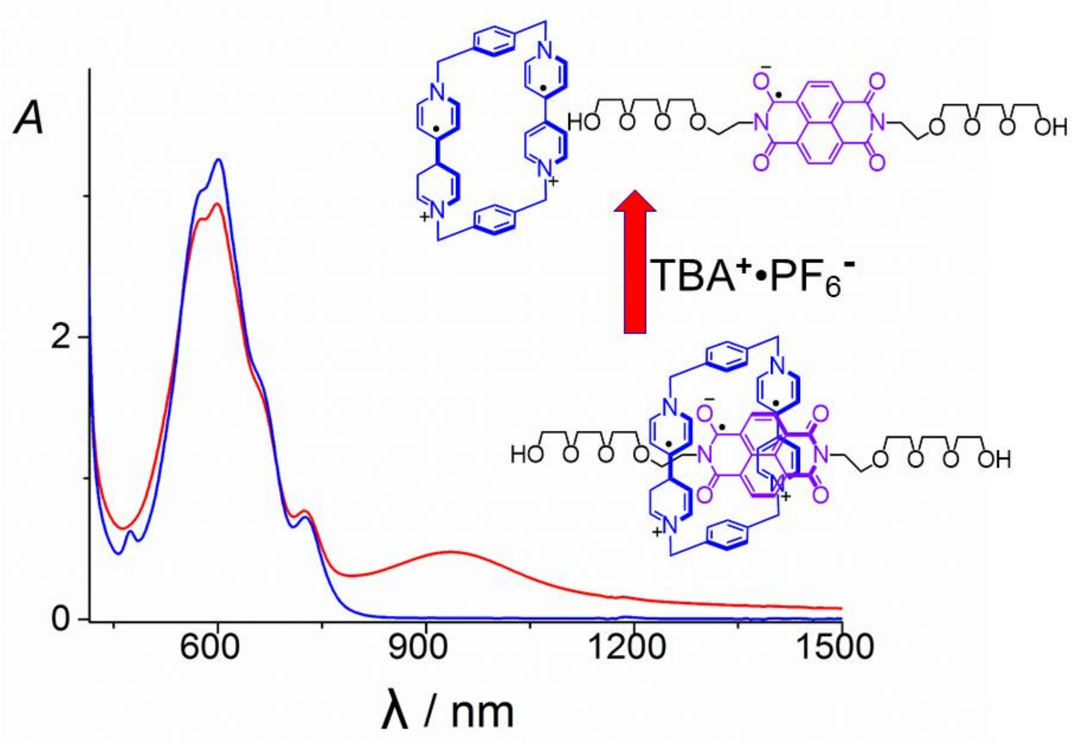

Supplementary Figure 18. The UV/Vis/NIR absorption spectra of 1:1 mixture of $\mathbf{2}^{-\bullet}$ (0.1 mM) and $CBPQT^{2(+\bullet)}$ before (red trace) and after (blue trace) **TBA**•PF_6 was added into the solution.

We also observed that $\mathbf{2}^{-\bullet}\subset\mathbf{CBPQT}^{2(+\bullet)}$ complex underwent dissociation in the presence of concentrated electrolyte, namely tetrabutylammonium

hexafluorophosphate (**TBA**$^{+}$•4PF$_6^{-}$). Upon addition of **TBA**•4PF$_6$ (500 mM) into the solution of **2**$^{-•}$⊂**CBPQT**$^{(+•)}$ in MeCN, the borad absorption band centered on 937 nm (Supplementary Figure 18, red trace) disappeared (Supplementary Figure 18, blue trace). This observation indicates that the complexation between **2**$^{-•}$ and **CBPQT**$^{(+•)}$ relies on both radical pairing interactions and Coulombic attraction of two opposite charges. The latter interaction was disrupted in the presence of highly concentrated electrolyte.

The 1:1 binding model of **2**$^{-•}$⊂**CBPQT**$^{2(+•)}$ complex was established by employing UV/Vis/NIR absorption spectroscopy (Supplementary Figure 19). The UV/Vis/NIR absorption spectra of the mixture of **2**$^{-•}$ and **CBPQT**$^{2(+•)}$ at different molarity ratios are recorded (Supplementary Figure 19a), when the overall concentrations of these two compounds remained constant (0.2 mM). A job plot, A_{937nm} vs the X_{host} was obtained (Supplementary Figure 19b), in which A_{937nm} represents the absorption of the spectrum at 937 nm for each mixture, and X_{host} represents the percentage of the molarity (i.e., [**CBPQT**$^{2(+•)}$]/([**CBPQT**$^{2(+•)}$]+ [**2**$^{-•}$])) of the host in the mixture. When X_{host} = 0.5, A_{937nm} gets the maximum value, which indicates that **CBPQT**$^{2(+•)}$ and **2**$^{-•}$ bind in a 1:1 manner.

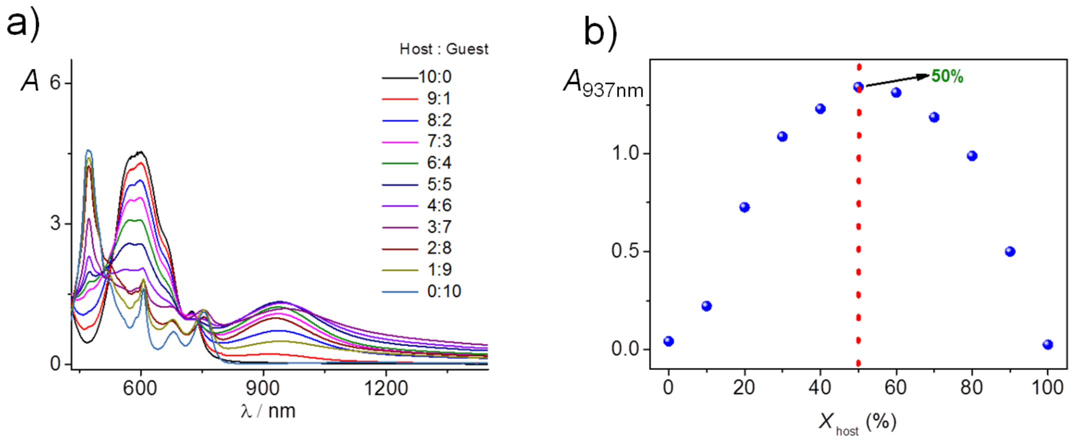

Supplementary Figure 19. a) The UV/Vis/NIR absorption spectra (298 K) of mixtures of **CBPQT**$^{2(+•)}$ and **2**$^{-•}$ at different molar ratio. [**CBPQT**$^{2(+•)}$] + [**2**$^{-•}$] = 0.2 mM. The absorbance of 937 nm, $A_{937\ nm}$, is employed to make the job plot in b).

We then employed UV/Vis absorption spectroscopy (Supplementary Figure 20a) to estimate the binding constant of the $\mathbf{2}^{-\bullet}\subset\mathbf{CBPQT}^{2(+\bullet)}$ complex, by using $\mathbf{2}^{-\bullet}$ to titrate the solution of $\mathbf{CBPQT}^{2(+\bullet)}$ in MeCN. The following equation is used to calculate the binding constant:

$$y = \frac{(1+1/K[C] + x) - ((1+1/K[C] + x)^2 - 4x)^{1/2}}{2/a}$$

In this equation, *[C]* represents the concentration of $\mathbf{CBPQT}^{2(+\bullet)}$, which was kept constant in all spectra during titration; *x* represents the molarity ratio of the guest $\mathbf{2}^{-\bullet}$ to the host $\mathbf{CBPQT}^{2(+\bullet)}$, namely *x* = [Guest] / [Host]; *y* represents the absorption at the maximal absorption wavelength, namely 890 nm; *K* represents the binding constants of $\mathbf{2}^{-\bullet}\subset\mathbf{CBPQT}^{2(+\bullet)}$; *a* is the maximal absorption at 890 nm, which is a constant. The binding constant for the formation of $\mathbf{2}^{-\bullet}\subset\mathbf{CBPQT}^{2(+\bullet)}$ complex was calculated (Supplementary Figure 20b) from a plot of the absorption at 937 nm versus [Guest]/[Host], namely $[\mathbf{2}^{-\bullet}]/[\mathbf{CBPQT}^{2(+\bullet)}]$. From this plot, a binding constant K_a = $1.2 \pm 0.6 \times 10^5$ M^{-1} could be deduced. A free energy of binding ΔG therefore is calculated to be around −7 kcal mol^{-1}, by using the equation $\Delta G = -RT\ln K_a$.

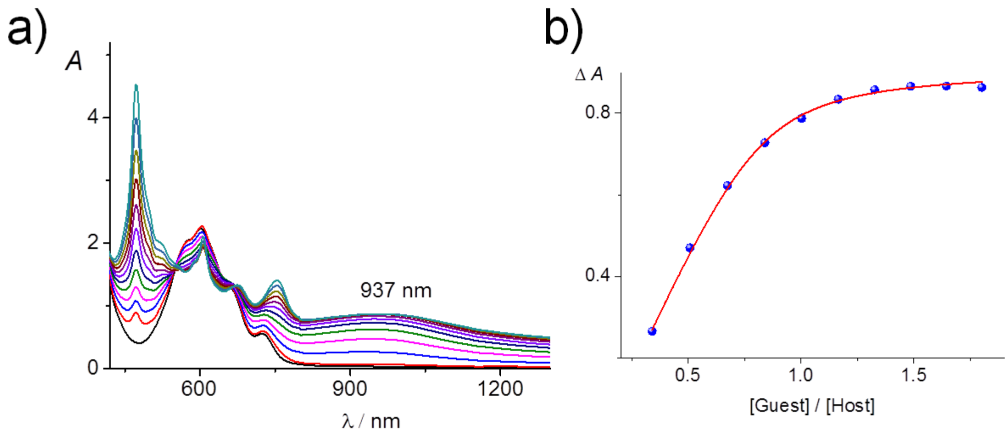

Supplementary Figure 20. a) The UV/Vis/NIR absorption spectra the mixture of $\mathbf{2}^{-\bullet}$ and $CBPQT^{2(+\bullet)}$ at different molar ratio in MeCN at 298 K, in order to calculate the binding constant of the supramolecular complex $\mathbf{2}^{-\bullet}\subset\mathbf{CBPQT}^{2(+\bullet)}$. The absorption at 937 nm is employed to make the job plot in b). The concentration of $CBPQT^{2(+\bullet)}$ is unchanged (1.2×10^{-4} M) during the titration process.

It is noteworthy that inaccuracy might exist in the evaluation of both binding constant K_a and ΔG. This is because trace amount of oxygen always exists in the system, which will oxidize **CBPQT**$^{2(+•)}$ to its oxidized form, namely **CBPQT**$^{4+}$. The consequence is that, using **2**$^{–•}$ to titrate **CBPQT**$^{2(+•)}$ always begins with a redox process, namely, **2**$^{–•}$ + **CBPQT**$^{4+}$ →**CBPQT**$^{2(+•)}$ + **2**. As a result, inaccuracy might exist in the evaluation of binding constant inaccurate.

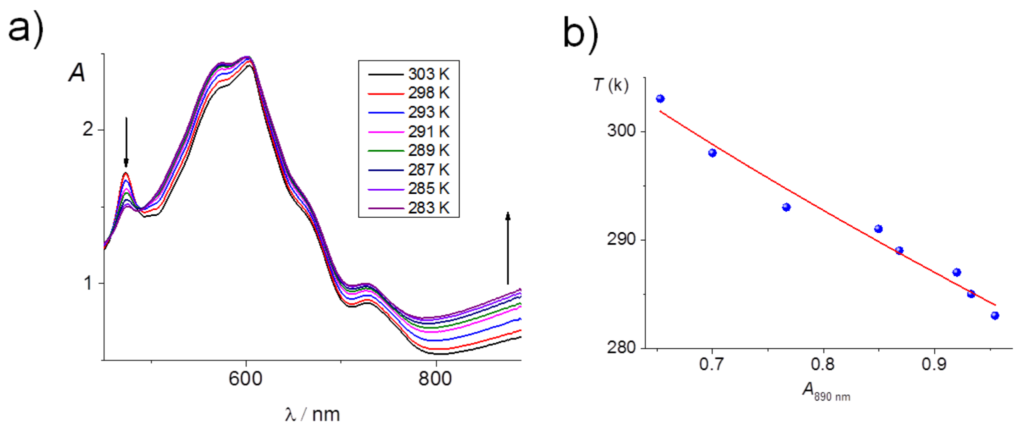

Supplementary Figure 21. a) The UV/Vis absorption spectra the 1:1 mixture of **2**$^{–•}$ and **CBPQT**$^{2(+•)}$ at variable temperatures between 283 and 303 K in MeCN. The absorptions at 890 nm in each spectra are employed to make the job plot in b). The concentrations of **2**$^{–•}$ and **CBPQT**$^{2(+•)}$ are both 1.0 × 10$^{–4}$ M.

In order to evaluate the ΔH and ΔS in the formation of **2**$^{–•}$⊂**CBPQT**$^{2(+•)}$, the UV/Vis spectra of the 1:1 mixture of **2**$^{–•}$ and **CBPQT**$^{2(+•)}$ at variable temperatures was recorded.

	**2**$^{–•}$ +	**CBPQT**$^{2(+•)}$ ⇌	**2**$^{–•}$⊂**CBPQT**$^{2(+•)}$
Concentrations in the begin (M)	C_0	C_0	0
Concentrations after the equilibrium (M)	$(1\text{-}a)C_0$	$(1\text{-}a)C_0$	aC_0

For a 1:1 mixture of **2**$^{–•}$ and **CBPQT**$^{2(+•)}$, the initial concentrations of both **2**$^{–•}$ and **CBPQT**$^{2(+•)}$ are C_0. After the occurrence of host-guest complexation, if the concentration of the complex is aC_0 (a represents the percentage of the host and guest that take part in the complexation), the concentrations of **2**$^{–•}$ and **CBPQT**$^{2(+•)}$ should be both $(1–a)C_0$, in which a is number between 0 and 1.

The binding constant K_a = [$\mathbf{2}^{-\bullet}$⊂$\mathbf{CBPQT}^{2(+\bullet)}$]/([$\mathbf{2}^{-\bullet}$][$\mathbf{CBPQT}^{2(+\bullet)}$]) = $a/((1-a)^2C_0)$.

Because the absorption (x) of the spectra at 937 nm of the 1:1 mixture of $\mathbf{2}^{-\bullet}$ and $\mathbf{CBPQT}^{2(+\bullet)}$ is proportional to the concentration of the complex $\mathbf{2}^{-\bullet}$⊂$\mathbf{CBPQT}^{2(+\bullet)}$, the following equation could be obtained: $x = Aa$

When a =1, x=A. This indicates that A is the maximal absorption, when all the host and guest undergo complexation to form the complex $\mathbf{2}^{-\bullet}$⊂$\mathbf{CBPQT}^{2(+\bullet)}$. Then the following equation could be obtained.

$$\Delta G = -RT\ln K_a = -RT\ln\frac{a}{(1-a)^2C_0} = -RT\ln\frac{x/A}{(1-x/A)^2C_0}$$

Because ΔG = ΔH − TΔS, the following equation could be obtained by combining the former two equations.

$$\Delta H - T\Delta S = -RT\ln\frac{x/A}{(1-x/A)^2C_0}$$

Then the relationship between the temperature T and the absorption at 937 nm could be deducted in the following equation:

$$T = \frac{\Delta H}{\Delta S - R\ln\frac{x/A}{(1-x/A)^2C_0}}$$

ΔH and ΔS for the formation of $\mathbf{2}^{-\bullet}$⊂$\mathbf{CBPQT}^{2(+\bullet)}$ complex were calculated (Supplementary Figure 21b) from a plot of the temperatures versus the absorption at 937 nm of the 1:1 mixture of $\mathbf{2}^{-\bullet}$ and $\mathbf{CBPQT}^{2(+\bullet)}$, which are −7.6 ±9.1 kcal mol^{-1} and −8 ±24 cal mol^{-1}K^{-1}, respectively.

In order to rule out the possibility that the absorption bands in the NIR region that were observed in the spectra of $\mathbf{2}^{-\bullet}$⊂$\mathbf{CBPQT}^{2(+\bullet)}$ or $\mathbf{3}^{(+\bullet)(-\bullet)}$ result from cobaltocene, or

its oxidized cationic form, namely cobaltocenium, we recorded (Supplementary Figure 22) the UV/Vis/NIR spectra of the cobaltocene in the absence and presence of $FeCl_3$. The latter compound is used as an oxidant, which could oxidize cobaltocene to generate cobaltocenium. We discovered that all of these species, including cobaltocene, cobaltocenium, as well as Fe^{3+}, do not have absorption bands in the NIR region.

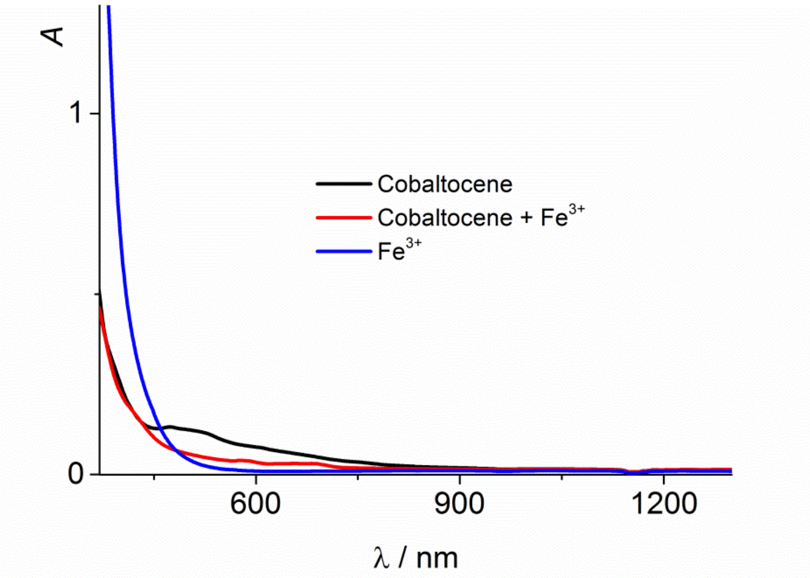

Supplementary Figure 22. The UV/Vis absorption spectra of cobaltocene (black trace), $FeCl_3$ (blue trace), as well as their 1:1 mixture in MeCN. The concentrations of cobaltocene and $FeCl_3$ are both 5.0×10^{-4} M, in all of these three solutions.

6. The characterization of the [2]Rotaxane 5^{4+}•$4PF_6^-$

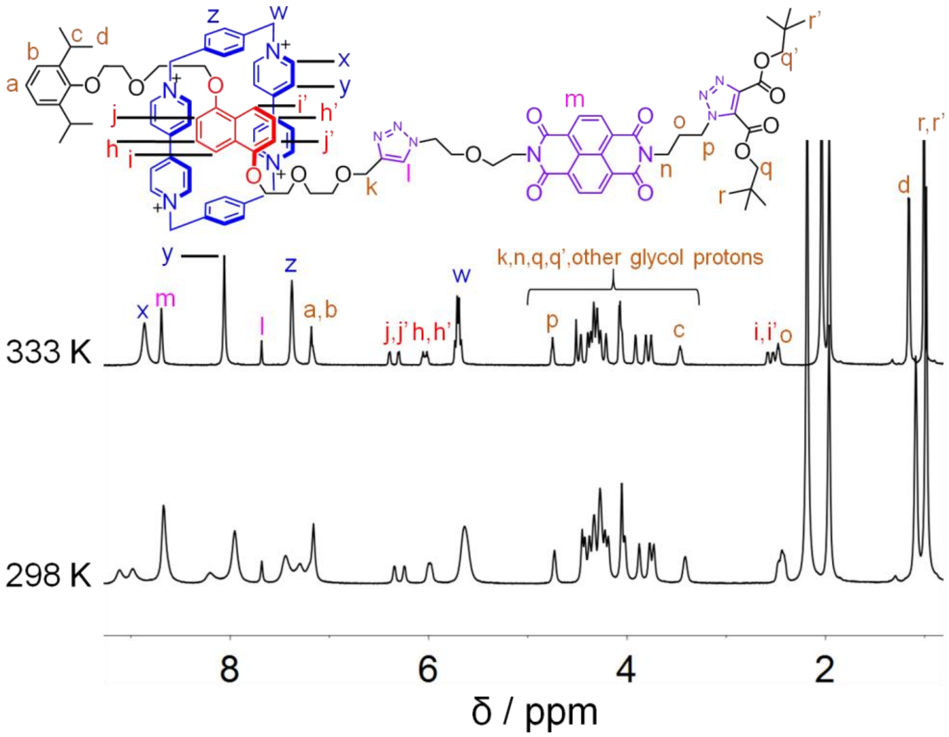

Supplementary Figure 23. ^{1}H NMR spectrum (600MHz, CD_3CN) the [2]rotaxane $\mathbf{5}^{4+}$•$4PF_6^-$ at 25 °C (bottom) and 60 °C (top).

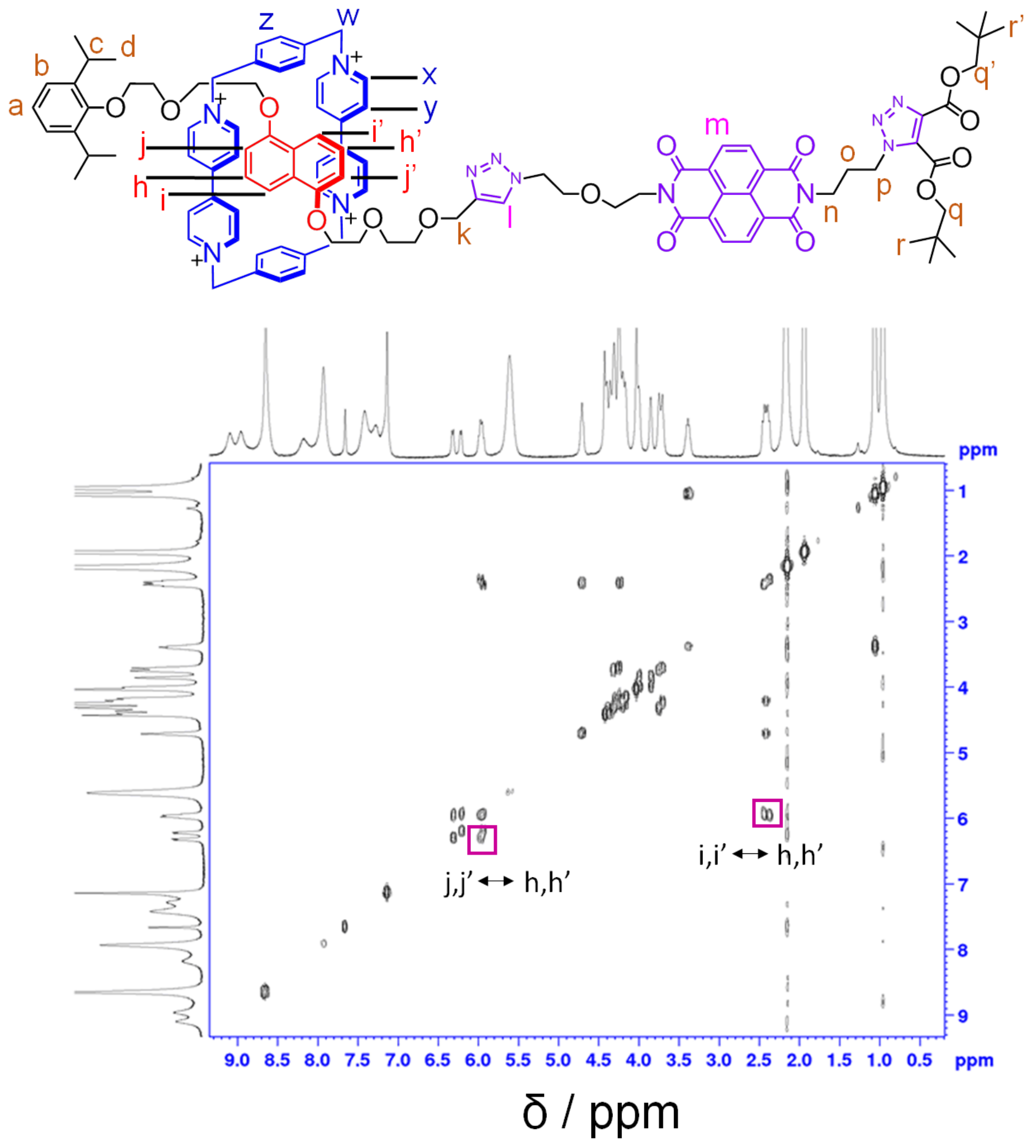

Supplementary Figure 24. ^1H–^1H Gradient-selected double-quantum filtered phase-sensitive COSY spectrum of the [2]rotaxane $\mathbf{5}^{4+}{\bullet}4PF_6^-$ (400MHz, CD_3CN, 298 K). The key correlation peaks corresponding to the **DNP** unit encircled by the ring are labeled in the spectrum.

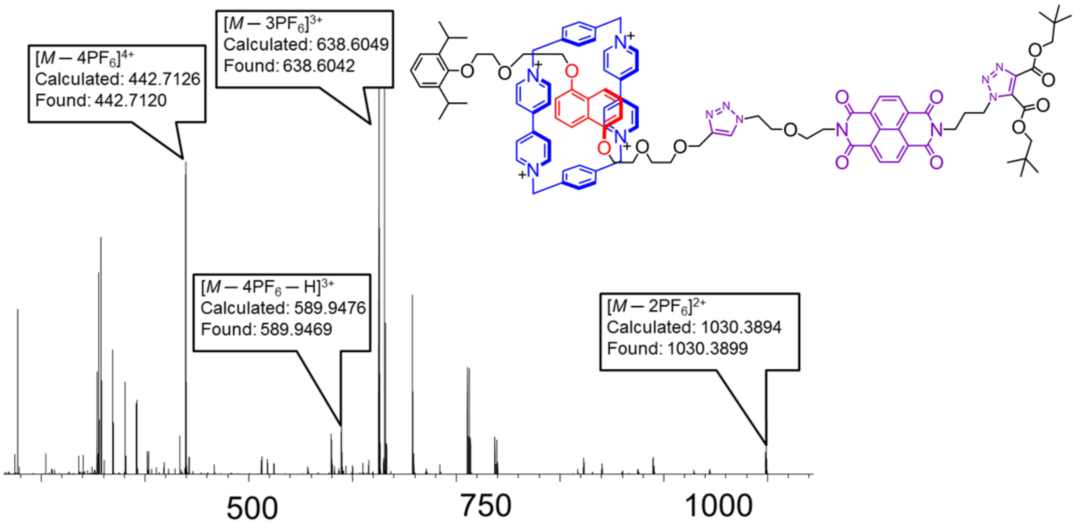

Supplementary Figure 25. ESI-MS of the [2]rotaxane **5**$^{4+}$•4PF_6^{-}. The signal labeled in the spectra correspond to molecular cations that contain four, three, and two positive charges.

7. Computational calculations.

All the calculations were performed using Gaussian 09 software package.[5] M06-2X[6,7] functional was used with 6-311G(d,p) basis set for all the atoms. The structures were optimized in the self-consistent reaction field (SCRF) using the SMD implicit solvent model[8] to evaluate the solvent effects in acetonitrile. Spin Unrestricted calculations were applied to all molecules and complexes with unpaired electrons. The vibrational frequencies of the optimized stationary points are calculated under the same level of theory, to obtain the zero-point vibrational energy (ZPVE) and thermal corrections at 298 K as well as verifying whether each optimized stationary points is an energy minimum. The 3D diagrams of optimized structures were generated using CYLView.[9] For the illustration of intrinsic interactions between NDI$^{-•}$ and CBPQT$^{2(+•)}$, the molecular orbitals were calculated using Gaussian 09 and visualized with Multiwfn[10,11] and VMD.[12]

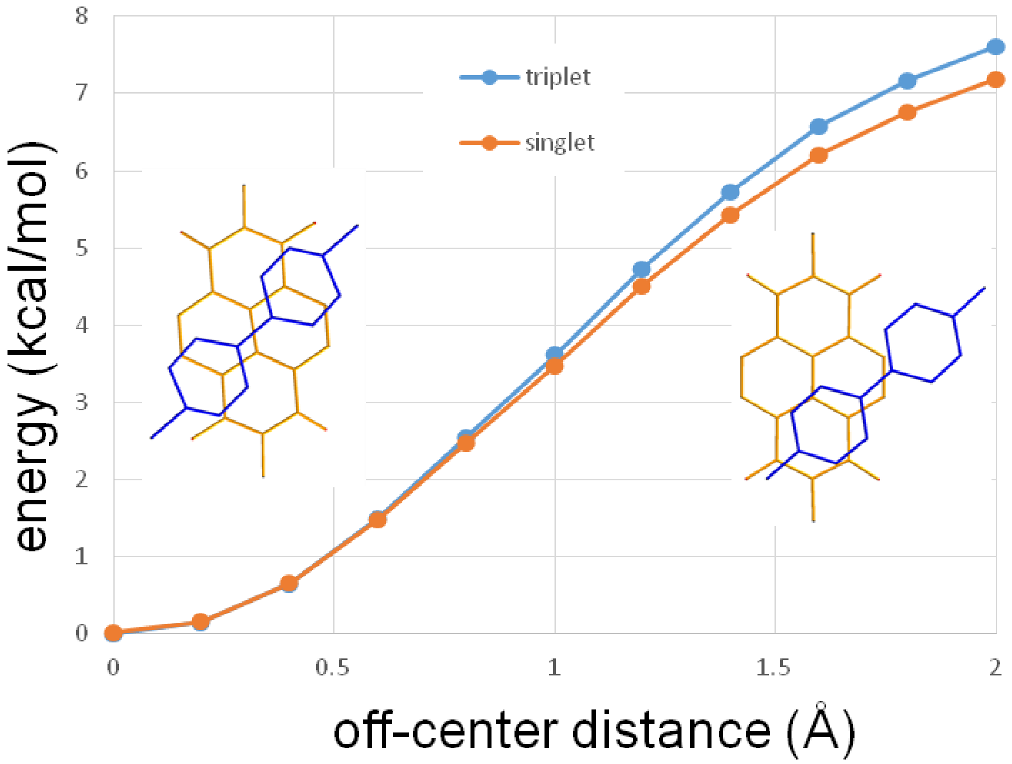

Supplementary Figure 26. DFT-calculated binding energy (ΔH) of the **BPY**$^{+\bullet}$/**NDI**$^{-\bullet}$ radical pair in its singlet (orange plot) and triplet (blue plot). These results demonstrate that when the two radicals are center aligned, the triplet-singlet energy gap is remarkably small, and the spin-spin interactions are very weak. Increasing the off-center distance of these two radical moieties, the triplet-singlet energy gap becomes larger, and favors spin-spin interactions between **BPY**$^{+\bullet}$ and **NDI**$^{-\bullet}$.

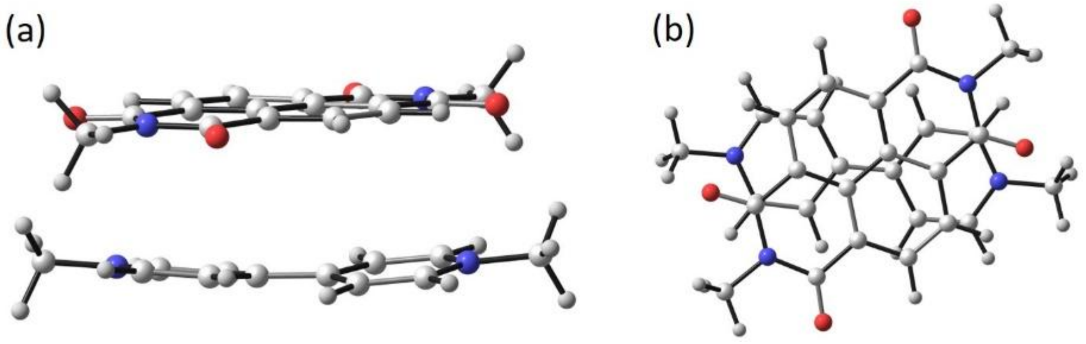

Supplementary Figure 27. DFT-optimized structures of **BPY**$^{+\bullet}$/**NDI**$^{-\bullet}$ (triplet) radical pair (a) side view and (b) top view.

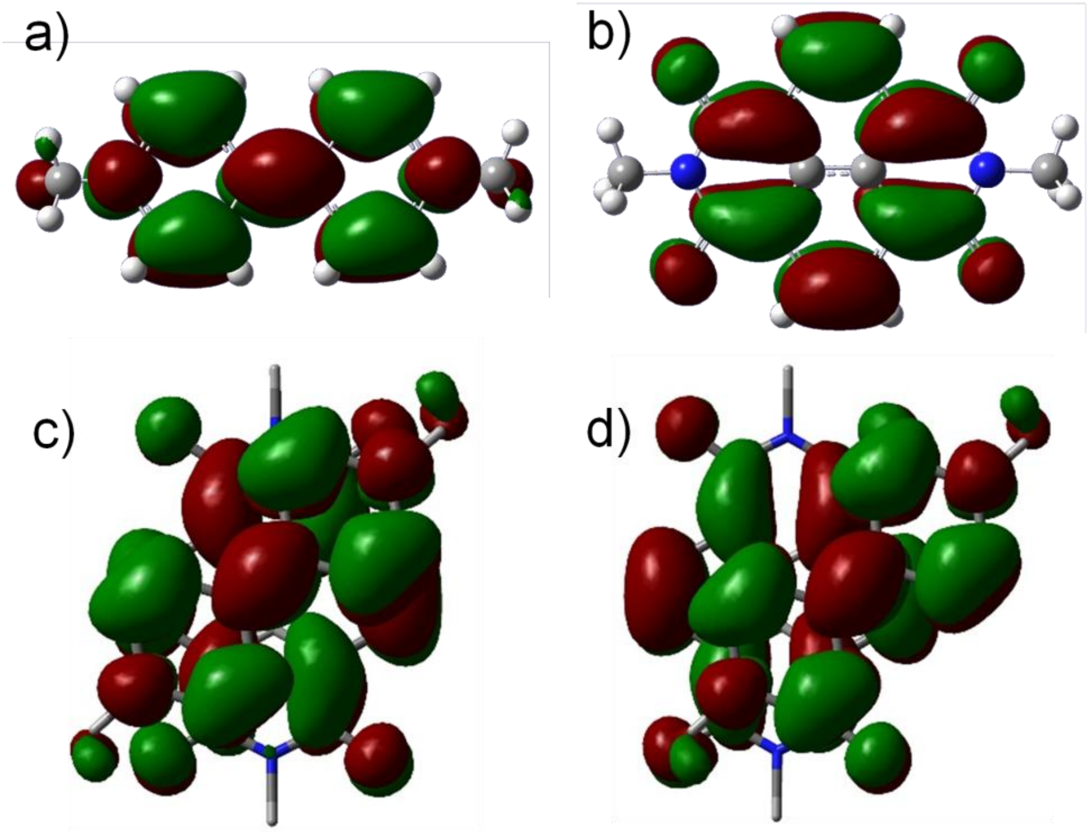

Supplementary Figure 28. DFT-calculated SOMOs of a) **BPY**$^{+\bullet}$ radical cation, which is symmetric in the molecular plane b) **NDI**$^{-\bullet}$ radical anion, which is antisymmetric in the molecular plane c) the center-aligned **BPY**$^{+\bullet}$/**NDI**$^{-\bullet}$ radical pair and d) a parallel displaced **BPY**$^{+\bullet}$/**NDI**$^{-\bullet}$ radical pair. For c) and d), both alpha and beta orbitals are shown. Because the symmetry-mismatched SOMOs of the **BPY**$^{+\bullet}$ radical cation and the **NDI**$^{-\bullet}$ radical anion, spin-spin interactions only occur in the case of parallel displaced stacking.

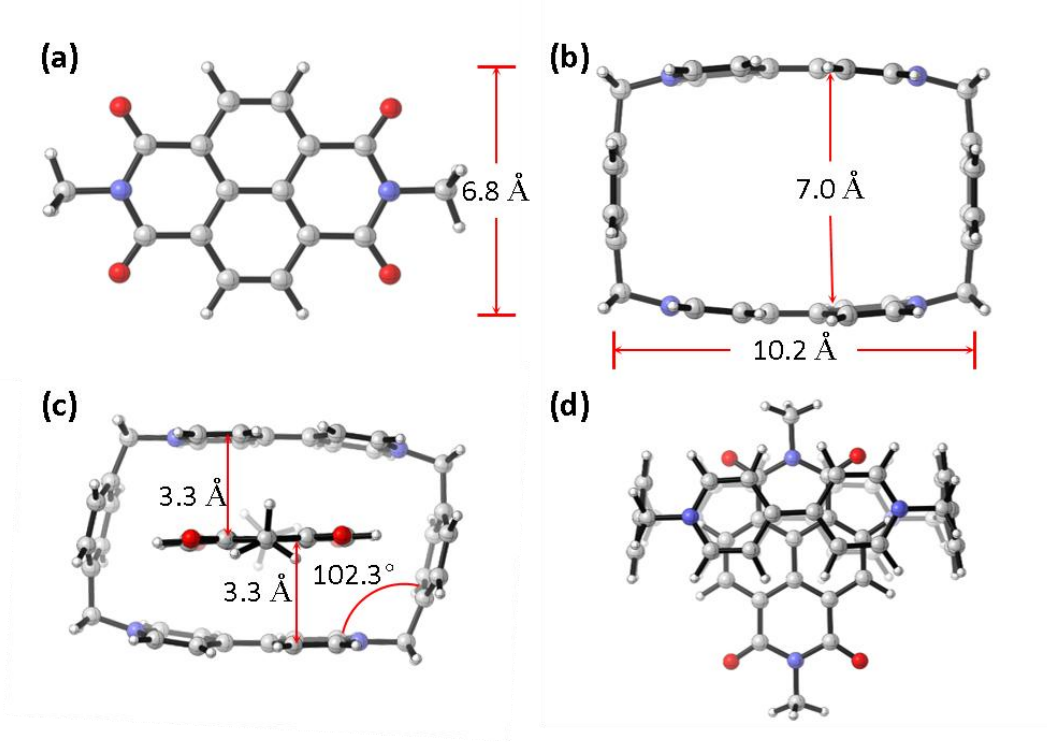

Supplementary Figure 29. DFT-optimized structures of **NDI**$^{-\bullet}$ (dimethyl substituted) (a), (b) **CBPQT**$^{2(+\bullet)}$ and the **NDI**$^{-\bullet}$⊂**CBPQT**$^{2(+\bullet)}$ complex, including (c) side view and (d) top view.

The thermodynamic parameters, including energies, enthalpies, and free energy of the calculated structures were summarized in Table S1.

**Structures**	***ZPE***	*ΔH*	*ΔG*	***E***	***H***	***G***	$<s^2>$
**NDI**$^{-\bullet}$⊂**CBPQT**$^{2(+\bullet)}$	0.842306	0.891398	0.764357	-2635.827565	-2634.936167	-2635.063208	0.8616
**CBPQT**$^{2(+\bullet)}$ (triplet)	0.607173	0.638369	0.545502	-1609.549049	-1608.910679	-1609.003547	2.0005
**NDI**$^{-\bullet}$	0.234444	0.252426	0.188628	-1026.247559	-1025.995133	-1026.058931	0.7503
**BPY**$^{+\bullet}$	0.240286	0.253804	0.199819	-574.693645	-574.692701	-574.746687	0.7654
**BPY**$^{+\bullet}$/**NDI**$^{-\bullet}$ (triplet)	0.475836	0.507590	0.413284	-1600.713835	-1600.712890	-1600.807197	2.0340
**BPY**$^{+\bullet}$/**NDI**$^{-\bullet}$ (singlet)	0.475819	0.507576	0.414346	-1600.713835	-1600.712891	-1600.806121	1.0344

Supplementary Table 1. Zero-point correction (ZPE), thermal correction to enthalpy (Δ*H*), thermal correction to Gibbs free energy (Δ*G*), energies (*E*), enthalpies (*H*), Gibbs free energies (*G*) (in Hartree), and $<S^2>$ values (after annihilation) calculated at the M06-2X/6-311G(d,p)-SMD (acetonitrile) level of theory.

Supplementary Reference

1. Barnes, J. C. *et al*. Synthesis of Ex^{n} Box Cyclophanes. *J. Org. Chem.* **78**, 11962-11969 (2013).

2. Bell, Toby DM, *et al*. Melt-induced fluorescent signature in a simple naphthalenediimide. *Chem. Commun*. **46**, 4881-4883 (2010).

3. Trabolsi, A. *et al*. Radically enhanced molecular recognition. *Nat. Chem*. **2**, 42-49 (2010).

4. Wu, Y. *et al*. Electron delocalization in a rigid cofacial naphthalene-1,8:4,5-bis(dicarboximide) dimer. *Angew. Chem. Int. Ed*. **53**, 9476-9481 (2014).

5. Frisch, M. J. T. *et al. Gaussian 09, revision C.01;* . Gaussian Inc.: Wallingford, CT, 2010.

6. Zhao, Y. & Truhlar, D. G., Density functionals with broad applicability in chemistry. *Acc Chem Res*. **41**, 157-167 (2008).

7. Zhao, Y. & Truhlar, D. G., The M06 suite of density functionals for main group thermochemistry, thermochemical kinetics, noncovalent interactions, excited states, and transition elements: two new functionals and systematic testing of four M06-class functionals and 12 other functionals. *Theor. Chem. Acc.* **120**, 215-241 (2008).

8. Marenich, A. V. *et al*. Universal solvation model based on solute electron density and on a continuum model of the solvent defined by the bulk dielectric constant and atomic surface tensions. *J. Phys. Chem. B* **113**, 6378-6396 (2009).

9. Legault, C. Y., *CYLView* **2009,** *1.0b*, http://www.cylview.org.

10. Lu, T. *et al*. Quantitative analysis of molecular surface based on improved Marching Tetrahedra algorithm. *J. Mol. Graphics Modell.* **38**, 314-323 (2012).

11. Lu, T. *et al*. Multiwfn: a multifunctional wavefunction analyzer. *J. Comput. Chem*. **33**, 580-592 (2012).

12. Humphrey, W. *et al*. VMD: Visual molecular dynamics. *J. Mol. Graphics*. **14**, 33-38 (1996).
